# *In vivo* epidermal migration requires focal adhesion targeting of ACF7

**DOI:** 10.1038/ncomms11692

**Published:** 2016-05-24

**Authors:** Jiping Yue, Yao Zhang, Wenguang G. Liang, Xuewen Gou, Philbert Lee, Han Liu, Wanqing Lyu, Wei-Jen Tang, Shao-Yu Chen, Feng Yang, Hong Liang, Xiaoyang Wu

**Affiliations:** 1 Ben May Department for Cancer Research, The University of Chicago, 929 East 57th Street, Chicago, Illinois 60637, USA; 2 State Key Laboratory Cultivation Base for the Chemistry and Molecular Engineering of Medicinal Resources, Ministry of Science and Technology of China, Guanxi Normal University, Guilin 541004, China; 3 Department of Pharmacology and Toxicology, University of Louisville Health Science Center, Louisville, Kentucky 40292, USA

## Abstract

Turnover of focal adhesions allows cell retraction, which is essential for cell migration. The mammalian spectraplakin protein, ACF7 (Actin-Crosslinking Factor 7), promotes focal adhesion dynamics by targeting of microtubule plus ends towards focal adhesions. However, it remains unclear how the activity of ACF7 is regulated spatiotemporally to achieve focal adhesion-specific guidance of microtubule. To explore the potential mechanisms, we resolve the crystal structure of ACF7’s NT (amino-terminal) domain, which mediates F-actin interactions. Structural analysis leads to identification of a key tyrosine residue at the calponin homology (CH) domain of ACF7, whose phosphorylation by Src/FAK (focal adhesion kinase) complex is essential for F-actin binding of ACF7. Using skin epidermis as a model system, we further demonstrate that the phosphorylation of ACF7 plays an indispensable role in focal adhesion dynamics and epidermal migration *in vitro* and *in vivo.* Together, our findings provide critical insights into the molecular mechanisms underlying coordinated cytoskeletal dynamics during cell movement.

## Abstract (web summary)

The spectraplakin protein ACF7 binds to actin at focal adhesions and targets microtubule plus ends to focal adhesions, promoting their disassembly. Here the authors reveal that ACF7 is phosphorylated by Src/FAK, and this regulates actin binding and focal adhesion dynamics *in vitro* and *in vivo*.

## Graphical abstract

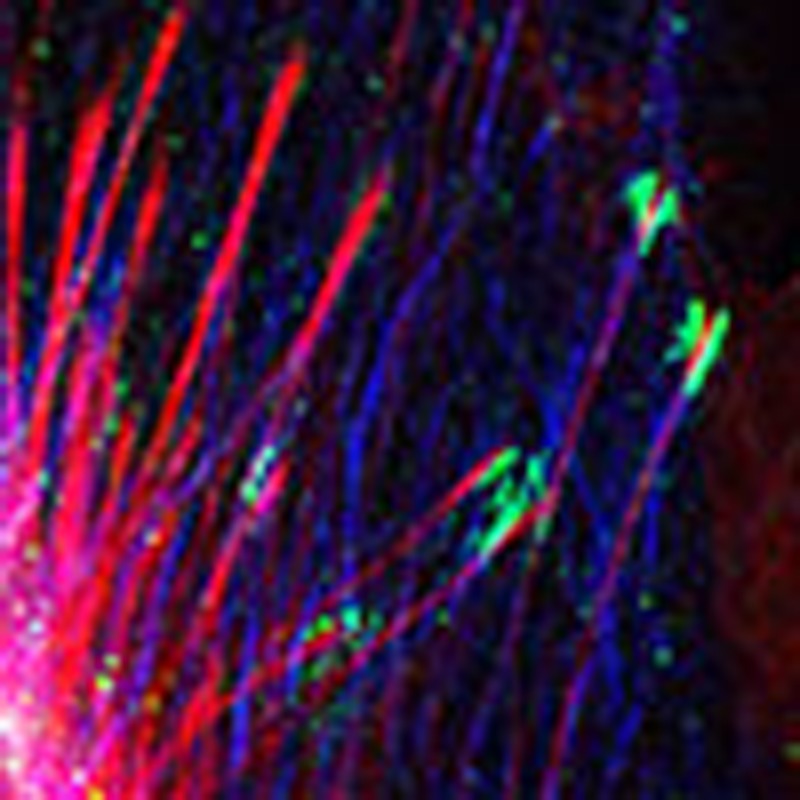

Cell migration is a fundamental cellular process. The multi-step process of cell migration entails integrated activities of the cytoskeletal networks and cell/extracellular matrix (ECM) adhesions^1^,^2^. Turnover of focal adhesions, organelles that connect the cytoskeletal network and ECM, is a critical component of this process. Interestingly, cytoskeletal dynamics coordinated by mammalian spectraplakin proteins have been shown to regulate focal adhesion dynamics during cell movement^3^,^4^.

Evolutionarily conserved in multicellular organisms, spectraplakin proteins such as ACF7 (Actin-Crosslinking Factor 7) can crosslink microtubules and F-actin^5^,^6^. Although broadly expressed, spectraplakins’ functions are best characterized in muscle, neurons and skin epidermal cells. Mutations in the single *Drosophila* spectraplakin gene leads to various defects that include perturbations in cytoskeletal organization, cell-cell junction and integrin-mediated epidermal attachment to muscle^7^. Mammalian genome encodes two spectraplakins, *BPAG1* (*Bullous Pemphigoid Antigen 1*) and *ACF7*. *BPAG1* mutant mice display sensory neuron and muscle degeneration phenotype, and have gross defects in cytoskeletal organization and function^7^. By contrast, ablation of *ACF7* expression in mice leads to early-embryonic lethality^8^,^9^. Conditional knockout (KO) of *ACF7* in mice suggest that ACF7 is critically involved in cytoskeletal coordination and cell migration *in vivo*^3^,^10^,^11^,^12^,^13^,^14^.

While the accumulating evidence underscores the importance of ACF7 in coordinating the cytoskeletal dynamics essential for cell movement, the spatiotemporal regulation of ACF7 remains largely unclear. ACF7 harbours an actin-binding domain (ABD) at its N terminus and a microtubule-binding domain at its C terminus^7^. The ABD of ACF7 contains two consecutive CH (calponin homology) domains^3^,^15^. In this study, we resolved the crystal structure of ACF7-NT. Our structural analysis indicated a closed conformation of the tandem CH domains in ACF7-NT, with key actin-binding sites (ABS) hidden by the intramolecular interactions^16^. Consistent with the structure results, endogenous ACF7 exhibits F-actin associated localization only at focal adhesions. This localization is apparently necessary for ACF7 to specifically guide growth of microtubule plus ends towards focal adhesions. The localization pattern also strongly suggests that cell-adhesion signalling is required for relieving the potential intradomain interactions within the ABD to allow ACF7 interaction with F-actin. However, the molecular nature of the signalling circuitry that regulates F-actin interaction of ACF7 and its localization at focal adhesions remains unknown.

ECM adhesion signalling is mediated by integrin receptors^17^. Although they have relatively short cytoplasmic domains, integrins can transmit the adhesion signals through their coupling to signalling molecules, such as tyrosine kinases. In this setting, the linked activities of two non-receptor tyrosine kinases, FAK (focal adhesion kinase) and Src family protein kinases, serve as a common intracellular point of convergence in the signalling network initiated by integrin receptors^18^. Both FAK KO cells and cells deficient in the three most ubiquitously expressed Src family kinases exhibit a decreased rate of migration and impaired focal adhesion turnover^19^,^20^,^21^. Although the connection between FAK/Src complex and cytoskeletal systems has been documented, it is still largely unknown how this complex may affect the coordination of microtubules and F-actin dynamics to regulate cell adhesions.

In this study, we identify a key tyrosine-phosphorylation site within the ABD of ACF7. Our results show that the FAK/Src-mediated phosphorylation of ACF7 is essential for F-actin binding of ACF7 and coordinated cytoskeletal dynamics at focal adhesions. By disrupting this site, we demonstrate that phosphorylation plays a key role in focal adhesion dynamics and cell motility *in vitro* and *in vivo.* Taken together, our results illuminate an important molecular mechanism whereby cell adhesion and cytoskeletal coordination are regulated during directional cell movement.

## Results

### ACF7 regulates epidermal migration *in vivo*

**Figure 1:**
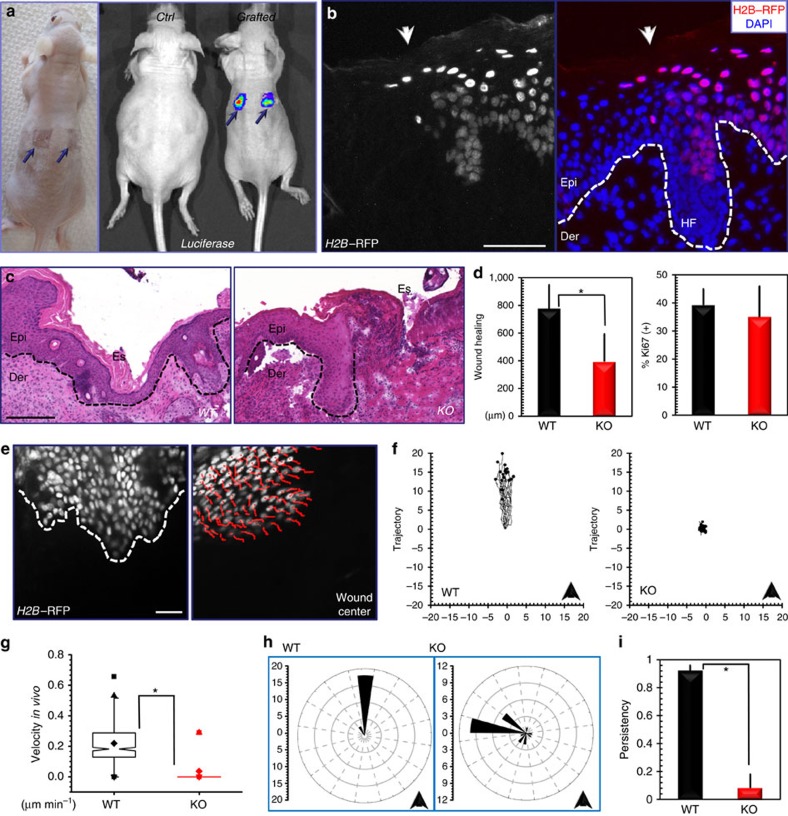
ACF7 regulates epidermal migration *in vivo*. (**a**) Image of nude mouse grafted with organotypic skin culture (left panel). Arrows denote grafted skin. Intravital imaging shows efficient incorporation of grafted cells expressing luciferase (arrows) on engraftment (right panel). (**b**) Sections of nude skin with grafted H2B–RFP-expressing keratinocytes were stained with DAPI. Dotted lines denote dermal–epidermal boundaries. Arrow heads denote the boundary between grafted skin and host skin. Scale bar, 50 μm. Epi, epidermis; Der, dermis; HF, hair follicle. (**c**) Wound healing as monitored by histological staining of skin sections at the wound edges 6 days after injury. Halves of wound sections are shown. Dotted lines denote dermal–epidermal boundaries. Wounding will induce proliferation and migration of epidermal keratinocytes, which lead to formation of hyperproliferative epidermis (HE) at the wound edge. Note significantly longer HE in the WT grafted skin comparing with the KO counterpart. Es, eschar. Scale bar, 100 μm. (**d**) Quantification of the length of HE generated at times indicated after wounding (left panel). Error bars represent s.e.m. *P<0.05 (student *t*-test). The right panel shows the quantification of Ki67-positive cells present in wound HE. Error bars represent s.d. The difference is not statistically significant (*P*=0.58). (**e**) Intravital imaging of H2B-GFP expression skin cells with multiphoton microscopy. The dashed line denotes wound edge. The red lines in the right panel show migration trajectory of each cell. Scale bar, 50 μm. (**f**) Plotting of individual epidermal cell-movement trajectories *in vivo.* The arrowhead indicates the direction towards wound centre. (**g**) Box and whisker plots of cell velocities *in vivo.* The plot indicates the mean (solid diamond within the box), 25th percentile (bottom line of the box), median (middle line of the box), 75th percentile (top line of the box), 5th and 95th percentile (whiskers), 1st and 99th percentile (solid triangles) and minimum and maximum measurements (solid squares). *P<0.05 (student *t*-test). (**h**) Windrose plotting of cell-movement directions. The arrow head indicates the direction towards wound centre. (**i**) Quantification of cell-movement persistency *in vivo.* Error bar represents s.d. *P<0.05 (student *t*-test).

Although approaches to exploring cell migration *in vitro* are readily available, it remains challenging to develop mammalian models and examine cell movement *in vivo*. As a surface organ, mammalian skin serves as an ideal candidate for intravital monitoring of cell migration. In addition, the established protocol for culturing skin epidermal stem cells makes it possible to generate functional skin tissue from engineered stem cells, which can carry both fluorescence markers for intravital imaging and defined genetic alterations to investigate the consequence in epidermal migration *in vivo.* However, engraftment of passaged epidermal stem cells via traditional skin stem cell technique proved unsuccessful. To resolve this issue, we first employed organotypic culture of epidermal keratinocytes *in vitro* by culturing the cells on top of acellularized dermis (Supplementary Fig. 1A). Exposure to the air/liquid interphase can induce stratification of cultured cells to generate a skin-like organoid *in vitro* (Supplementary Fig. 1B) (ref. 22). Transplantation of this cultured skin organoid to nude host leads to efficient skin engraftments, which are stable and can readily express exogenous genes that have been transduced to the epidermal stem cells (Fig. 1a,b). The grafted skin displayed stratification and differentiation programme indistinguishable from the host skin (Supplementary Fig. 1C).

Our previous studies have suggested a role of ACF7 in cell motility and skin-wound repair^3^,^14^. When grafted skins were challenged to respond to injury, skin generated from *ACF7*-deficient cells exhibited a significant delay in repairing full-thickness wounds as compared with wild-type (WT) skin (Fig. 1c). Histological analysis and quantification revealed that the area of hyperproliferative epidermis (HE) that typically proliferates and migrates into the wound site was diminished by >50% at day 6 following injury in skin grafted with *ACF7* null cells (Fig. 1d). Despite the difference in wound closure, no significant alterations were found in cell proliferation in grafted skins after wounding, as assessed by labelling for Ki67 (Fig. 1d).

To directly monitor epidermal migration *in vivo*, we transduced WT and *ACF7* null cells with lentivirus encoding *Histone H2B–RFP*. Regenerated skins were wounded and then imaged by multiphoton microscopy (Supplementary Movies 1 and 2). WT skin exhibited robust and well-polarized movement of epidermal cells, whereas most *ACF7* KO cells showed little or no movement during the same time interval (Fig. 1e,f). Quantification of these movements revealed a dramatic decrease in average speed *in vivo* on loss of *ACF7* (Fig. 1g). In addition, loss of *ACF7* greatly compromised polarity of cell movement, as indicated by both windrose plotting of cell-movement angles and quantification of movement directionality (Fig. 1h,i). Epidermal cells migrate in a collective manner in response to wounding^23^. Consistent with this notion, WT cells migrate *in vivo* with significant uniformity as indicated by Rayleigh test (*P*=1.44 × 10^−7^). By contrast, *ACF7* null cells move in a rather uncoordinated manner (*P*=0.516 by Rayleigh test). Taken together, our results demonstrate that ACF7 plays an essential and multifaceted role in epidermal migration *in vivo.*

### Crystallographic analysis of ACF7-NT domain

**Figure 2:**
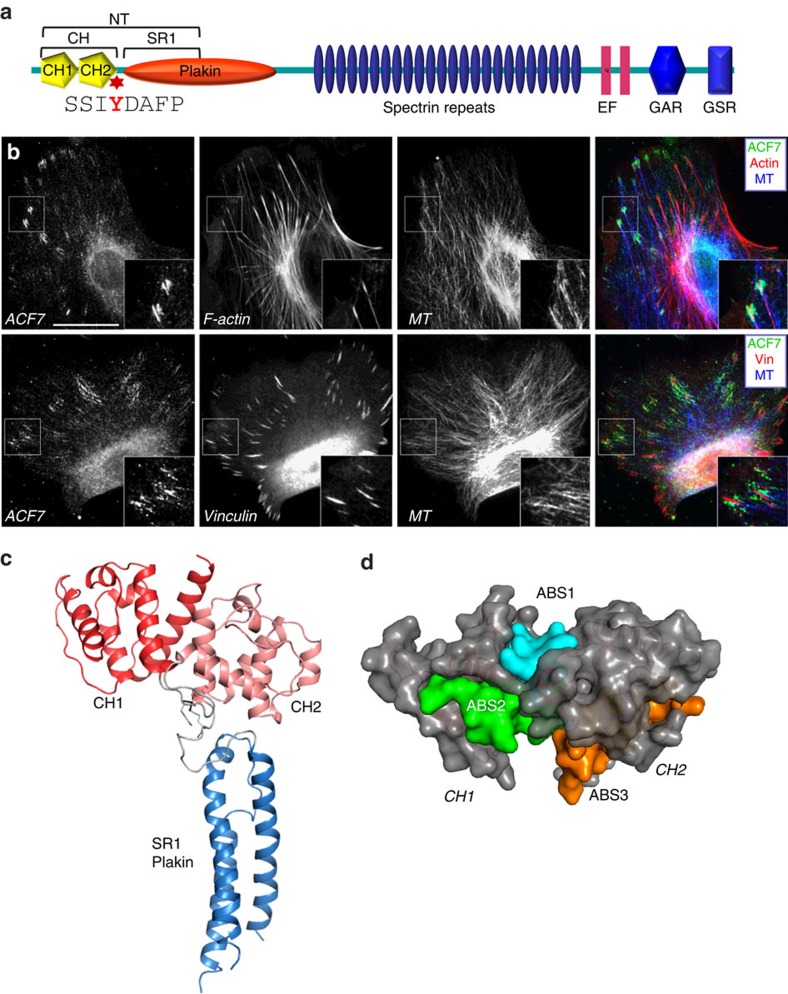
Crystal structure of ACF7-NT. (**a**) Diagram of ACF7 domain structure. The following abbreviations are used: CH, calponin homology domain; SR1, spectrin repeat 1 (of Plakin domain); EF, EF hand motif; GAR-GSR, Gas2 related and GSR repeats for microtubule-binding domain. The second CH domain in the N terminus contains the tyrosine-phosphorylation site (phosphor-tyrosine in red). (**b**) Immunofluorescence for ACF7 (green), F-actin or focal adhesions/vinculin (red), and microtubules (blue) shows enriched ACF7 staining at the tips of actin fibres and focal adhesions. Boxed areas are magnified as insets. Scale bar, 20 μm. (**c**) ACF7-NT structure. Two tandem CH domains and the SR1 of Plakin domain are marked. (**d**) The three potential F-actin-binding sites (ABS1–3) were partially hidden in the closed conformation of tandem CH domains in ACF7’s ABD. ABS1, cyan; ABS2, green; and ABS3, orange.

ACF7 is a microtubule and F-actin crosslinker that is critically involved in regulation of cell/ECM adhesion dynamics^7^. However, it remains unclear how ACF7 can achieve specific cytoskeletal crosslinking to mediate microtubule targeting to focal adhesions. ACF7 harbours the ABD (the tandem CH domains) and a plakin domain in its N terminus (Fig. 2a). Although the ABD domain has F-actin-binding affinity *in vitro*^15^, ACF7 does not exhibit extensive co-localization with F-actin filaments in cells (Fig. 2b). Instead, in primary mouse keratinocytes, endogenous ACF7 are enriched at the plus tips of microtubules that associate only with the ends of actin stress fibres, where focal adhesions localize (Fig. 2b). The localization pattern is consistent with the role of ACF7 in mediating specific targeting of microtubule plus ends towards peripheral focal adhesions, through its actin and microtubule crosslinking activity.

The two tandem CH domains are present in ABDs of many F-actin-binding proteins^16^. Based on known structures of ABDs, the CH domains usually adopt a closed conformation (Supplementary Fig. 2A). This conformation is achieved by an extensive interdomain interaction interphase, causing key ABS to be largely buried within the two CH domains. Thus, it has been proposed that the exposure of ABS requires potential domain movement/rearrangement of CH domains to allow interaction with F-actin^16^. Previous studies suggest that different ABDs associate with F-actin in different manners, and significant domain movement has been demonstrated for CH domain-containing proteins, such as utrophin^24^,^25^,^26^,^27^. However, it remains unclear how ACF7’s CH domains associate with F-actin.

To illuminate the potential mechanisms of how F-actin binding of ACF7-CH domains is regulated, we resolved crystal structure of ACF7-NT (containing the CH domains and a part of the plakin domain) at 2.6 Å resolution (Fig. 2c, Supplementary Fig. 2A–C, and Table 1). ACF7-NT is folded into an elongated polypeptide that is ∼92.4-Å long and ∼58/14.3-Å wide with two tandem CH domains following the spectrin repeat 1 (SR1) of the plakin domain. The SR1 of ACF7’s plakin domain consists of three α-helices connected by short loops that pack in a left-handed helical bundle with a typical up–down–up topology, which is highly conserved among plakin family protein^28^,^29^. ACF7’s ABD consists of only α-helices connected by short-loop sequences, and adopts a closed conformation with extensive intramolecular contacts between the two tandem CH domains (Fig. 2c,d and Supplementary Fig. 2A). Previous biochemical and mutagenesis studies have revealed three major F-ABSs (ABS1–3) in the tandem CH domains^16^, which are conserved in ACF7 (Supplementary Fig. 2D). However, in closed conformation of ACF7’s ABD, a number of key residues in the ABSs were buried within the protein and were unlikely to be involved in association with F-actin (Fig. 2d).

Previous studies indicate that CH domains of utrophin associate with F-actin through an induced-fit mechanism, requiring a transition to an extended conformation between the tandem CH domains on binding with F-actin^16^,^26^,^30^. Considering enrichment of ACF7 at focal adhesions, our results suggest that cell-adhesion signalling may enhance ACF7 association with F-actin by increasing the flexibility of ACF7’s CH domain.

### FAK/Src kinase complex phosphorylates ABD of ACF7

**Figure 3:**
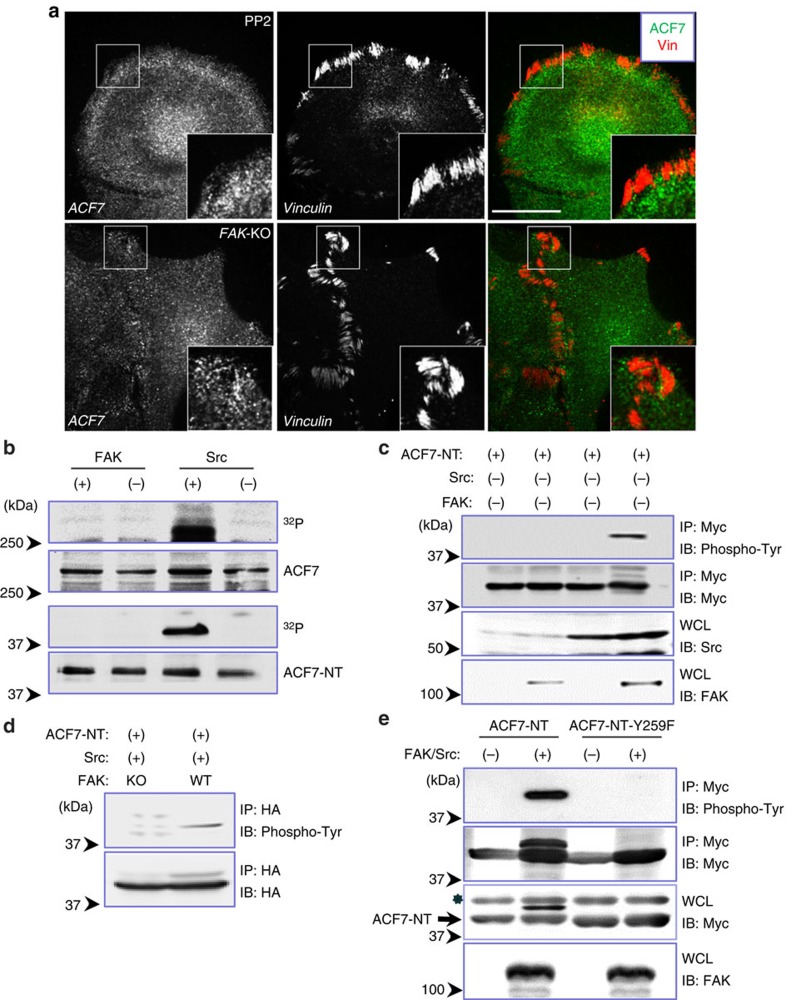
FAK and Src phosphorylate ACF7 at Y259. (**a**) Immunofluorescence for ACF7 (green) and focal adhesions (vinculin, red). Note decreased localization of ACF7 in focal adhesions in PP2-treated cells or cells deficient for *FAK*. Boxed areas are magnified as insets. Scale bar, 20 μm. (**b**) *In vitro* kinase assays were performed on full-length ACF7 or ACF7-NT with recombinant FAK or Src proteins. Phosphorylation was analysed by SDS–PAGE and autoradiography. (**c**) Lysates are collected from cultured HEK293 cells expressing Myc-tagged ACF7-NT together with FAK and/or Src. Lysates were immunoprecipitated with α-Myc antibody. Immunoprecipitates (IP) and aliquots of whole-cell lysates (WCL) were analysed by SDS–PAGE and immunoblotting (IB) with different antibodies as indicated. (**d**) WT or *FAK* KO keratinocytes were transfected with plasmids encoding myc-tagged ACF7-NT and Src. After immunoprecipiation, proteins were subjected to SDS–PAGE and immunoblotting with different antibodies as indicated. (**e**) Lysates are collected from cultured cells expressing Myc-tagged ACF7-NT or ACF7-NT-Y259F mutant together with or without FAK and Src. Lysates were analysed by SDS–PAGE on immunoprecipiation and immunoblotting with different antibodies as indicated. The arrow denotes the band of ACF7-NT or NT-Y259F in lysates, and the ‘*’ denotes a non-specific background band present in the lysate. Note phosphorylation of WT *ACF7* but not Y259F mutant leads to a band shift.

FAK and Src tyrosine kinases are the two most prominent signalling molecules that are activated by integrin receptors^18^. *In vivo*, FAK and Src form a mutually activated kinase complex that initiates a cascade of phosphorylation events, triggering various signalling pathways downstream to cell adhesion. In particular, both FAK and Src have been shown to be critically involved in cell motility and focal adhesion dynamics^19^,^20^,^21^. Interestingly, when we treated mouse keratinocytes with a Src inhibitor, PP2, ACF7 failed to localize at focal adhesions (Fig. 3a, quantification in Supplementary Fig. 3A). In addition, ACF7 localization at focal adhesions was greatly diminished in cells deficient for *FAK* (Fig. 3a, quantification in Supplementary Fig. 3A). Our results suggest that focal adhesion signalling may regulate ACF7 connection with F-actin through FAK/Src complex.

To investigate the link between ACF7 and FAK/Src complex, we examined the potential modification of ACF7 by FAK and Src tyrosine kinases. Kinase assays with purified recombinant proteins showed that Src but not FAK can efficiently phosphorylate full-length ACF7 and ACF7-NT *in vitro* (Fig. 3b). When expressed exogenously in cultured HEK293T cells, FAK or Src alone did not lead to efficient phosphorylation of ACF7-NT, as determined by immunoblots with phosphor-tyrosine-specific antibodies. Co-expression of both FAK and Src strongly phosphorylated ACF7-NT (Fig. 3c), suggesting that formation of a mutually activated kinase complex is required for efficient phosphorylation of ACF7 in cells. Consistent with this notion, expression of Src in WT keratinocytes, but not in FAK-deficient keratinocytes, leads to significant phosphorylation of ACF7-NT (Fig. 3d).

To determine the FAK/Src phosphorylation site within ACF7-NT, we next analysed phospho-ACF7-NT by mass spectrometry (MS). Liquid chromatography-tandem MS (LC–MS/MS) analysis identified a prominent phosphorylation site (Y259) within the 2nd CH domain at ACF7-NT (Fig. 2a and Supplementary Fig. 3B). To confirm our MS results, we replaced the predicted tyrosine site with phenylalanine and repeated our phosphorylation assays. The single mutation at Y259 completely abolished ACF7-NT phosphorylation by FAK and Src (Fig. 3e). Interestingly, the identified tyrosine residue is evolutionarily conserved (Supplementary Fig. 3C), suggesting a critical role for phosphorylation of Y259.

### Phosphorylation is essential for ACF7 binding with F-actin

**Figure 4:**
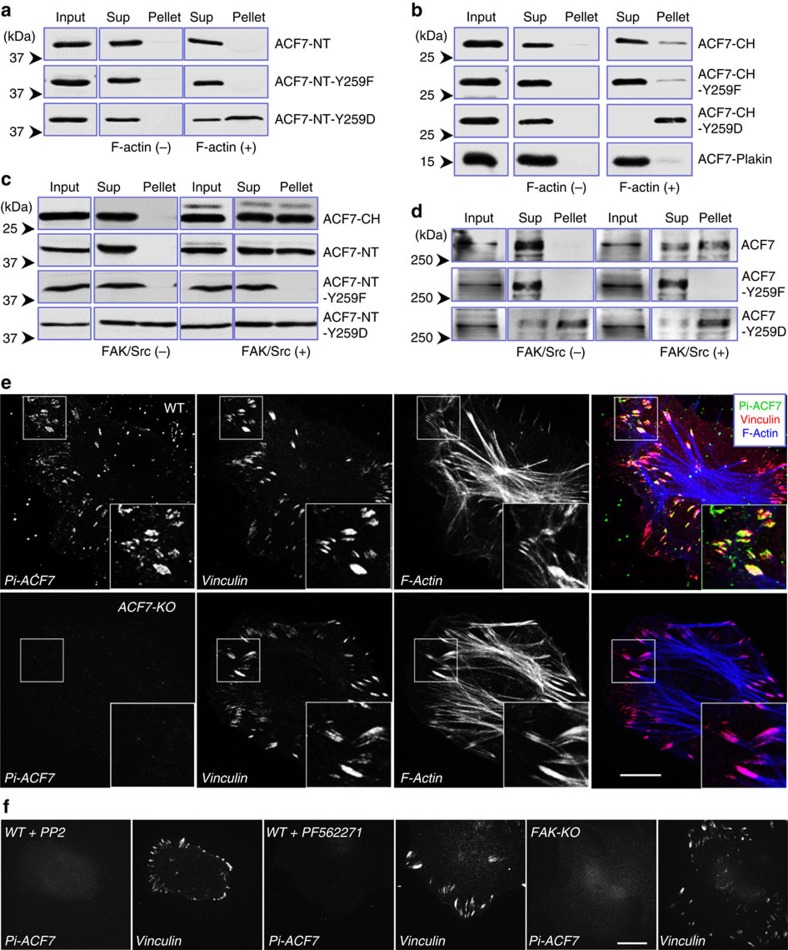
Phosphorylation of ACF7 at Y259 promotes F-actin binding. (**a**,**b**) ACF7-NT, CH, plakin fragments or their mutants on Y259 were isolated from *E*. *coli* as His-tagged recombinant proteins. F-actin binding was examined by co-sedimentation assay. Pellet and supernatant, as well as an aliquot of pre-cleared protein sample (input) were immunoblotted with α-His6 to determine F-actin-binding affinity. Sup, supernatant. (**c**) Cells were transfected with plasmids encoding *ACF7*-NT or NT mutants together with or without FAK or Src. F-actin binding was examined by co-sedimentation assay. Pellet and supernatant,as well as an aliquot of pre-cleared cell lysate (input) were immunoblotted to assess the level of ACF7. (**d**) Cells were transfected with plasmid encoding full-length *ACF7* or *ACF7* mutants together with or without FAK or Src. F-actin binding was examined by co-sedimentation assay. Pellet and supernatant, as well as an aliquot of pre-cleared cell lysate (input) were immunoblotted to assess the level of ACF7. (**e**) Immunofluorescence for focal adhesions (vinculin, red), phosphor-ACF7 (green) and F-actin (blue) shows specific localization of phosphor-ACF7 at focal adhesions in WT but not *ACF7 null* cells. Boxed areas are magnified as insets. Scale bar, 20 μm. (**f**) Immunofluorescence for focal adhesions and phosphor-ACF7 in *FAK* null cells or WT keratinocytes treated with Src inhibitor, PP2. Scale bar, 20 μm.

Our identification of the FAK/Src phosphorylation site in ACF7’s ABD suggest a potential role of this modification in regulation of ACF7’s binding with F-actin. To test this possibility, we expressed and purified ACF7-NT or CH domain (Fig. 2a), as well as their point mutants that converted the phosphorylation site to either a kinase-refractory version harbouring a tyrosine to phenylalanine mutation (Y259F mutant) or a phospho-mimetic version, containing a tyrosine to aspartic acid mutation (Y259D mutant). We then carried out *in vitro* co-sedimentation assays with polymerized F-actin to determine their binding affinity (Fig. 4a,b). Both ACF7-CH and its Y259F mutant displayed a weak but detectible binding with F-actin. By contrast, ACF7-NT and its Y259F mutant cannot be efficiently pulled down by F-actin under the same condition, suggesting that the presence of a plakin domain may play an inhibitory role in ACF7 association with F-actin. However, when phospho-mimetic mutation was introduced (Y259D), both ACF7-NT and CH exhibited robust interaction with F-actin *in vitro* (Fig. 4a,b).

To directly assess the effects of FAK/Src-mediated phosphorylation, we next co-expressed FAK and Src kinases with ACF7-CH or NT domain in cells. As predicted, the actin-binding affinity of ACF7 was greatly enhanced when FAK/Src is present (Fig. 4c). This effect was specific, as co-expression of FAK/Src did not alter F-actin interactions with ACF7-NT mutants (Y259F or Y259D) (Fig. 4c).

To determine whether the phosphorylation regulates F-action association of full-length ACF7, we engineered expression vectors encoding haemagglutinin (HA)-tagged full-length ACF7, as well as point mutants at Y259. As expected, full-length ACF7 or ACF7-Y259F mutant displayed very weak interaction with F-actin in co-sedimentation assay, whereas the phospho-mimetic mutation dramatically increased F-actin binding (Fig. 4d). In addition, co-expression of FAK/Src with full-length ACF7 greatly enhanced WT ACF7 binding with F-actin, but not the mutants (Fig. 4d).

Consistent with biochemical analysis, ACF7-NT or its Y259F mutants exhibited no significant decoration of F-actin filaments when expressed in primary keratinocytes. By contrast, Y259D mutant in ACF7-NT showed marked co-localization with F-actin (Supplementary Fig. 4A). Together, our biochemical and cell biology analysis presented compelling evidence that phosphorylation of Y259 promotes ACF7 association with F-actin, unveiling a hitherto unrecognized mechanism of CH domain interaction with F-actin.

To further investigate the role of ACF7 phosphorylation by FAK/Src, we generated a phospho-specific ACF7 antibody against the synthetic phospho-peptides corresponding to the Y259 site at the CH domain. The antibody was specific for the phosphorylated state of ACF7: when ACF7-NT was not phosphorylated or when the site was selectively mutated, the antibody failed to recognize the ACF7-NT protein (Supplementary Fig. 4B). The phosphor-specific antibody of ACF7 detected the expected sized band in immunoblots of cultured WT keratinocytes but not *ACF7* KO cells (Supplementary Fig. 4C). The blotting signal was sensitive to chemical inhibitors of Src and genetic ablation of *FAK* (Supplementary Fig. 4C), further confirming the specificity of the antibody.

With the phosphor-specific antibody, we next examined the endogenous localization of phosphorylated ACF7. In primary keratinocytes, phospho-ACF7 staining showed striking co-localization with focal adhesions (Fig. 4e). Unlike staining for total ACF7 (Fig. 2b), diffusive or punctate localization in cytoplasm is barely detectible in phosphor-ACF7 staining. The staining was specific for ACF7, as it was abolished in *ACF7* KO cells (Fig. 4e). In addition, no significant phosphor-ACF7 can be detected in FAK-deficient cells or cells treated with PP2 or FAK inhibitor (Fig. 4f). These findings are consistent with the phospho-dependent affinity of ACF7 for F-actin that we observed *in vitro* and reveal a remarkable correlation between FAK/Src-mediated phosphorylation of ACF7 and ACF7–actin connection at focal adhesions.

### Regulation of ACF7 binding with F-actin by phosphorylation

**Figure 5:**
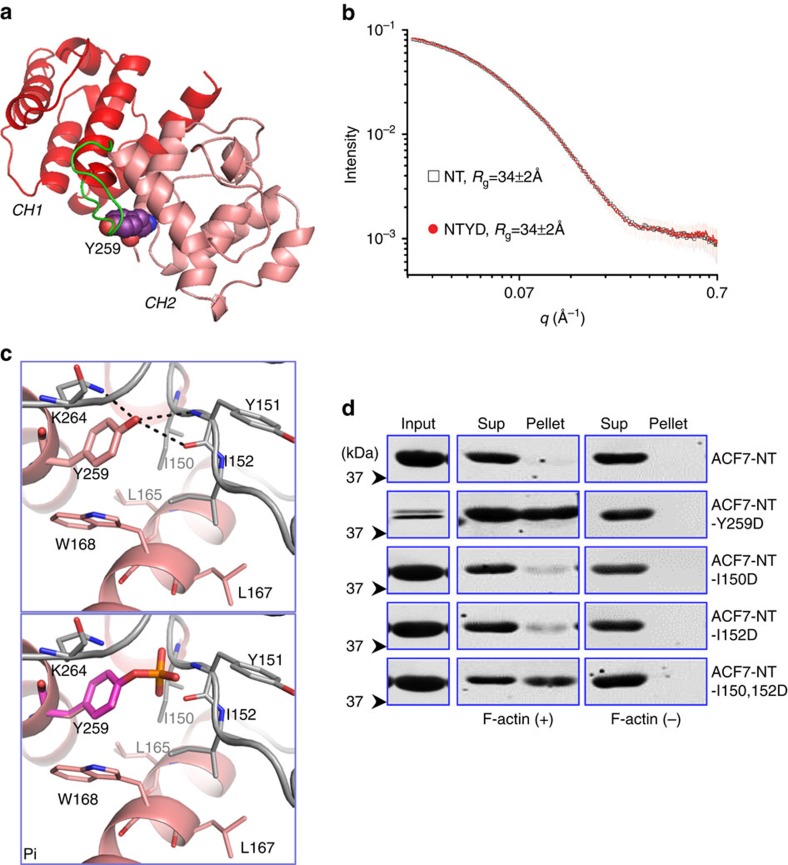
Structural basis underlying regulation of ACF7 binding with F-actin by phosphorylation of tyrosine 259. (**a**) Structure of ACF7’s ABD. Note the closed conformation of the tandem CH domains, and interaction between Tyrosine 259 and the loop between CH1 and CH2 (green). (**b**) SAXS scattering curves of ACF7-NT and NT Y259D in PBS. The Guinier *R*_g_ was calculated using PRIMUS in ATSAS package. Note the highly similar scattering curves of NT and NT mutant in solution. (**c**) Detailed view of ACF7-NT structure (upper panel). Note the close contact of tyrosine 259 with the hinge sequence between CH1 and CH2, and formation of multiple hydrogen bonds between tyrosine 259 and residues in the loop (dashed lines). Lower panel indicates predicted structure of ACF7-NT on phosphorylation of tyrosine 259. The phosphorylation will likely disrupt the hydrogen bonds, and the negatively charged phosphate group is projected towards the hinge sequence, which contains multiple hydrophobic residues. (**d**) ACF7-NT or its various mutants were isolated from *E*. *coli* as His-tagged recombinant proteins. F-actin binding was examined by co-sedimentation assay. Pellet and supernatant as well as an aliquot of pre-cleared protein sample (input) were immunoblotted with α-His6 to determine F-actin binding affinity.

The ABD of ACF7 adopts a closed conformation, with key ABSs being buried within the two CH domains (Fig. 2c,d). The two CH domains of ACF7 are connected with a short-loop sequence (Fig. 5a). Interestingly, tyrosine 259 is outside of the conserved ABSs (Supplementary Fig. 2B) and located at the C-terminal end of the last α-helix in the second CH2 domain. This residue is in close proximity with the hinge region of ABD in the closed conformation (Fig. 5a).

To determine how phosphorylation of tyrosine 259 might affect F-actin interaction, we determined in solution structure of ACF7-NT and Y259D mutant of ACF7-NT by small-angle X-ray scattering (SAXS). The gel filtration profiles of purified ACF7-NT and Y259D mutant suggest that both proteins are monomeric and monodisperse (Supplementary Fig. 5A–C). We found that the value of radius of gyration (*R*_g_=34 Å) derived from the SAXS data of ACF7-NT is slightly larger than that of the crystal structure of ACF7-NT (*R*_g_=29 Å), suggesting that ACF NT in solution is likely to be more extended than that depicted by the crystal structure (Supplementary Fig. 5D). Although ACF7-NT Y259D mutation exhibit strongly elevated F-actin-binding affinity, its SAXS scattering curve and pair distance distribution function (P(r)) are highly similar to the WT counterpart, suggesting that the mutant is structurally similar to WT protein without the ligand (F-actin) (Fig. 5b, and supplementary Fig 5E,F). Thus, instead of changing overall structure of ACF7’s ABD, phospho-mimetic mutation (Y259D) may enhance F-actin-binding affinity by affecting the intramolecular interactions within the tandem CH domains, allowing the dynamic flexibility of CH domains on F-actin interaction.

Structural analysis demonstrated that tyrosine 259 forms hydrogen bonds with isoleucine 150 and tyrosine 151 in the hinge region of CH domains (Fig. 5c). These hydrogen bonds will be disrupted on phosphorylation of tyrosine 259. In addition, the hinge region between CH1 and CH2 domain is stabilized by the hydrophobic interactions between isoleucine 150, isoleucine 152 and leucine 165, leucine 167 and tryptophan 168. When tyrosine 259 is phosphorylated, a strongly negatively charged phosphate group will be introduced, which will likely interfere in the hydrophobic interactions and thus enhance the flexibility of tandem CH domains in ACF7’s ABD. To explore this hypothesis, we introduced point mutations to the two isoleucine residues at the hinge region. Mutation of each individual isoleucine to aspartic acid leads to a small but appreciable increase in F-actin-binding affinity compared with WT ACF7-NT (Fig. 5d). When two isoleucine residues were mutated, the F-actin-binding affinity is markedly increased, to a level comparable with the Y259D mutant of ACF7-NT (Fig. 5d). Taken together, our results elucidate the potential structural basis for the mechanism by which ACF7 is regulated by cell-adhesion signalling to control its connection with F-actin.

### Phosphorylation is essential for focal adhesion dynamics

**Figure 6:**
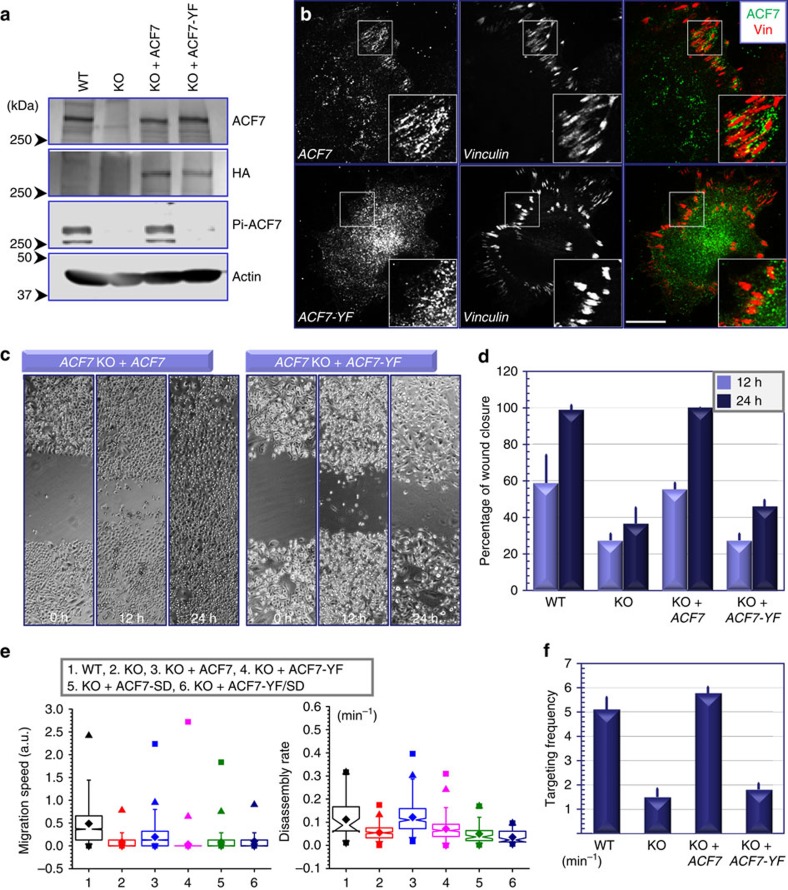
Phosphorylation of ACF7 on tyrosine 259 promotes focal adhesion dynamics and cell motility *in vitro*. (**a**) Lysates are collected from WT, *ACF7* KO and *ACF7* KO cells re-expressing *ACF7* or *ACF7*-Y259F mutant. Lysates were analysed by SDS–PAGE and immunoblotting with different antibodies as indicated. (**b**) Immunofluorescence for focal adhesions (vinculin, red) and exogenous *ACF7* (green) shows localization of WT but not Y259F mutant of *ACF7* at focal adhesions. Boxed areas are magnified as insets. Scale bar, 20 μm. (**c**) Confluent monolayers of keratinocytes with different genotypes were subjected to *in vitro* scratch-wound assays. Phase contrast images of the site were taken at hours (h) indicated after scratch-wounding. (**d**) Quantification of the kinetics of *in vitro* wound closure. Error bar represents s.d. One-way ANOVA indicates that the difference between WT versus KO, or KO versus KO+ACF7 or KO+ACF7 versus KO+ACF7-YF is statistically significant (*P*<0.05). (**e**) Box and whisker plots of cell velocities (left panel) and focal adhesion disassembly (right panel) in different keratinocyte cell lines as indicated. One-way ANOVA indicates that the difference between WT versus KO, or KO versus KO+ACF7, or KO+ACF7 versus KO+ACF7-YF, or KO+ACF7 versus KO+ACF7-SD or KO+ACF7 versus KO+ACF7-YFSD is statistically significant (*P*<0.05). (**f**) Behaviour of microtubule targeting to focal adhesions was monitored by confocal videomicroscopy and manually traced. Targeting frequencies to individual focal adhesions were quantified and depicted by bar graphs. Error bar represents s.d. One-way ANOVA indicates that the difference between WT versus KO, or KO versus KO+ACF7, or KO+ACF7 versus KO+ACF7-YF is statistically significant (*P*<0.05). ANOVA, analysis of variance.

Ablation of *ACF7* in skin keratinocytes impairs cell motility and focal adhesion dynamics^3^. To investigate the role of ACF7 phosphorylation in these processes, we constructed stable cell lines from *ACF7* KO keratinocytes to re-express *ACF7* or *ACF7* mutant. To achieve stable expression of *ACF7*, we overcame the technical hurdles of ACF7’s enormous size (5,380 amino acid residues) and engineered mammalian expression vectors encoding full-length *ACF7* or *ACF7*-Y259F mutant within PiggyBac (PB) transposon elements^31^. When introduced into KO keratinocytes together with PB transposase, the coding sequence for *ACF7* was efficiently integrated into the genomes. After selection, the cell lines expressed *ACF7* or its Y259F mutant stably. Exogenous *ACF7* and Y259F mutants were expressed comparably with the correct size, and exhibited the expected differential phosphorylation status (Fig. 6a). Exogenously expressed *ACF7* displayed focal adhesion-associated localization, reminiscent of the localization of endogenous ACF7 (Fig. 6b). By contrast, immunofluorescence staining showed more diffusive localization of *ACF7*-Y259F mutant in cytoplasm (Fig. 6b).

To determine the effect on cell motility, we first monitored the polarized rate of cell migration during recovery of ∼300 μm scratches introduced into keratinocyte monolayers. As shown previously^3^, while WT cells closed the gap within 24 h, KO keratinocytes usually moved only ∼50% of this distance into the wound (Fig. 6c,d). Re-expression of *ACF7*, but not its phosphorylation refractory mutant rescued the defect (Fig. 6c,d). Videomicroscopy permitted image and monitoring the velocities of individual keratinocytes. Assays of representative cells highlighted the significantly inhibited motility of KO cells as compared with WT keratinocytes as shown previously^3^. Exogenous expression of *ACF7*, but not its Y259F mutant in KO cells significantly restored cell motility (Fig. 6e).

Turnover of focal adhesion is a key step in cell movement, and ACF7 plays a pivotal role in this process^3^. To examine focal adhesion dynamics, we began by employing confocal videomicroscopy to trace the behaviour of individual focal adhesions^21^. To monitor the process, we transfected cells with plasmids encoding *DsRed-Zyxin*^9^,^32^. Quantifications of the kinetics of hundreds of individual focal adhesions revealed significant and marked decrease in the disassembly rates of focal adhesions in KO cells or KO cells rescued with Y259F mutant of *ACF7*, compared with WT cells or KO cells re-expressing full-length *ACF7* (Fig. 6e). Despite well-documented role of ACF7 in focal adhesion turnover, our previous report also demonstrates that ACF7 promotes assembly of nascent focal adhesions^3^. Directional movement of cells requires efficient assembly and disassembly of focal adhesions. With quantification of videomicroscopy results, our results indicate that WT *ACF7* but not its YF mutant can rescue the defect in focal adhesion assembly, suggesting that cytoskeletal crosslinking mediated by ACF7 plays an important role in focal adhesion formation (Supplementary Fig. 6).

Our previous results indicate that ACF7 connection with microtubules is regulated by GSK3β-mediated phosphorylation at the C-terminal domain of ACF7 (ref. 14). To test whether disruption of ACF7 interaction with both F-actin and microtubules may affect cell motility and focal adhesion dynamics, we prepared *ACF7* mutant that harbours Serine to Aspartic acid mutations at the GSK3β phosphorylation sites (ACF7-SD mutant) or both YF and SD mutations (ACF7-YF/SD mutant). When introduced into *ACF7*-deficient keratinocytes by microinjection, the SD mutant or YF/SD mutant of *ACF7* could not significantly rescue the defects in either cell motility or focal adhesion disassembly or assembly (Fig. 6e and Supplementary Fig. 6), providing compelling evidence that cytoskeletal crosslinking activity is essential for the role of ACF7 in these processes.

ACF7 promotes focal adhesion turnover by guiding microtubule plus ends towards focal adhesions^3^. To visualize microtubule plus-end movements and targeting to focal adhesions, we expressed GFP–EB1, DsRed-Zyxin and CFP-LifeAct (marker for F-actin)^33^ in cells. Consistent with our previous report^3^, confocal microscopy revealed that in KO keratinocytes rescued with WT *ACF7*, but not *ACF7*-Y259F mutant, the movements of microtubules plus-end were effectively guided towards focal adhesions along the underlying F-actin filaments (Supplementary Movies 3 and 4). With life cell imaging approach, we next quantified focal adhesion targeting frequency of microtubules in various cell lines. Our results indicate that loss of *ACF7* significantly reduces microtubule targeting towards focal adhesions, and expression of WT *ACF7* but not the phosphorylation refractory mutant restored focal adhesion targeting of microtubules in *ACF7* KO cells (Fig. 6f). Together, our results provide compelling evidence that phosphorylation of ACF7 by FAK/Src complex is essential for focal adhesion/cytoskeletal coordination and cell migration *in vitro.*

### ACF7 phosphorylation is critical for skin-wound repair *in vivo*

**Figure 7:**
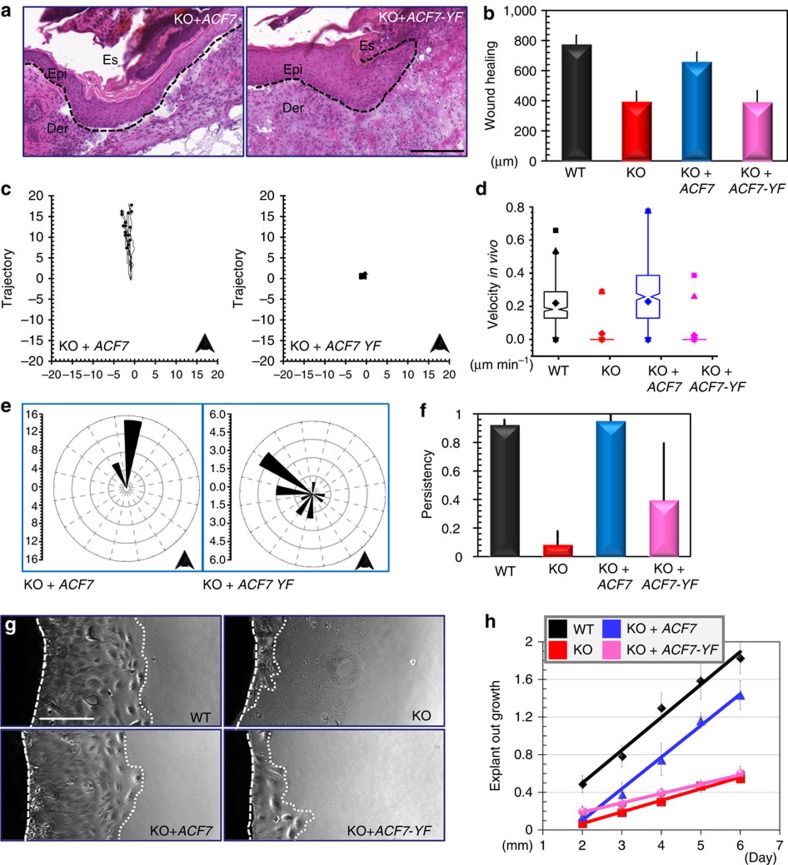
Phosphorylation of ACF7 by FAK and Src plays an essential role in skin-wound healing *in vivo*. (**a**) Wound healing as monitored by histological staining of skin sections at the wound edges 6 days after injury. Halves of wound sections are shown. Es, eschar. Dotted lines denote dermal–epidermal boundaries. Scale bar, 200 μm. (**b**) Quantification of the length of hyperproliferative epidermis generated at times indicated after wounding (upper panel). Error bars represent s.e.m. One-way ANOVA indicates that the difference between WT versus KO, or KO versus KO+ACF7 or KO+ACF7 versus KO+ACF7-YF is statistically significant (*P*<0.05). (**c**) Plotting of individual epidermal cell-movement trajectories *in vivo.* The arrow head indicates the direction towards wound centre. (**d**) Box and whisker plots of cell velocities *in vivo.* One-way ANOVA indicates that the difference between WT versus KO, or KO versus KO+ACF7 or KO+ACF7 versus KO+ACF7-YF is statistically significant (*P*<0.05). (**e**) Windrose plotting of cell-movement directions. The arrow head indicates the direction towards wound centre. (**f**) Quantification of cell-movement persistency *in vivo.* One-way ANOVA indicates that the difference between WT versus KO, or KO versus KO+ACF7 or KO+ACF7 versus KO+ACF7-YF is statistically significant (*P*<0.05). (**g**) Phase contrast images of skin-explant culture (day 4). Note outgrowth of epidermal cells is inhibited on loss of *ACF7*, which can be rescued by *ACF7* but not by *ACF7*-Y259F mutant. Dashed lines denote boundary of skin explant (left) or leading edge of epidermal outgrowth from the explant (right). Scale bar, 50 μm. (**h**) Quantification of epidermal outgrowth in skin explants. Error bar represents s.d. One-way ANOVA indicates that the difference between WT versus KO, or KO versus KO+ACF7 or KO+ACF7 versus KO+ACF7-YF is statistically significant (*P*<0.05). ANOVA, analysis of variance.

The skin engraftment platform provides an efficient way to examine the relevance of ACF7 phosphorylation *in vivo.* To determine the role of ACF7 phosphorylation in skin-wound repair, we engrafted *ACF7* KO rescued with WT *ACF7* or *ACF7*-Y259F mutant to nude mice. These cells were incorporated into host skin with comparable efficiency, and no significant difference was identified in cell proliferation or differentiation *in vivo. In vivo* re-expression of WT *ACF7*, but not Y259F mutant in the KO cells restored skin re-epithelialization (Fig. 7a,b).

To directly visualize epidermal migration *in vivo*, we transduced all the cells with lentivirus encoding *H2B–RFP*. Regenerated skins were wounded and imaged by multiphoton microscopy as shown before (Supplementary Movies 5 And 6). Quantification of these movements revealed that WT *ACF7* but not the phosphorylation refractory mutant can significantly rescue the defects in cell-migration speed and polarity (Fig. 7c–f). To further explore the role of ACF7 phosphorylation in skin re-epithelialization, we performed tissue explants *ex vivo*^34^. The biopsies of skin grafts were placed on fibronectin-coated glass dishes and monitored for the outgrowth of interconnected epithelial sheets of grafted keratinocytes from these explants (Fig. 7g). Quantification revealed a marked delay in the outgrowth from *ACF7* KO explants compared with WT counterparts. Exogenous expression of *ACF7* but not *ACF7*-Y259F mutant rescued the defect (Fig. 7h). Taken together, our studies presented strong evidence that phosphorylation of ACF7 by FAK/Src plays a critical role in epidermal migration during skin-wound repair *in vivo.*

## Discussion

The coordination of F-actin and microtubules plays an essential role in a variety of cellular processes^2^. Mounting evidence now suggests that focal adhesions can serve as the ‘hotspot’ for crosstalk between microtubules and F-actin cytoskeletal networks. It has been shown that microtubules can specifically grow towards focal adhesions, a process likely guided by underlying F-actin filaments^35^,^36^. Targeting of focal adhesions by microtubules promotes disassembly of focal adhesions, a feature in line with the long-standing observation that cells treated with microtubule-depolymerizing drugs contain enlarged and stabilized focal adhesions^3^,^37^,^38^. Our previous studies identified a microtubule and F-actin-crosslinking protein, ACF7, as an essential molecule in coordinating this process and promoting focal adhesion dynamics during directional cell movement^3^. Interestingly, loss of *ACF7* in mouse primary keratinocyte inhibits both assembly and disassembly of focal adhesions. Although it remains unclear how cytoskeletal crosslinking mediated by ACF7 can enhance focal adhesion assembly, establishing a focal adhesion-oriented microtubule network may facilitate specific delivery of effector proteins that may promote assembly and maturation of focal adhesions.

Despite its significant role in focal adhesion turnover, it remains unclear how the activity of ACF7 is regulated in the cells to achieve focal adhesion-specific guidance of microtubule. Our previous study revealed that microtubule-binding domain of ACF7 can be phosphorylated by protein kinase GSK3β, which leads to decreased microtubule-binding affinity and plays an important role to sustain directional movement of skin stem cells^14^. In addition, recent study has also demonstrated regulation of BPAG1n4 by calcium through the EF hand motif at the C terminus^39^.

In this study, by employing a combinatory approach encompassing structure biology, biochemistry and cell biology analysis, we present compelling evidence that cell-adhesion signalling can lead to phosphorylation of a key tyrosine residue at the second CH domain of ACF7 through the FAK/Src kinase complex. This posttranslational modification can greatly enhance ACF7 interaction with F-actin by potentially weakening the intramolecular interactions within tandem CH domains that block F-actin association. Consistent with the critical role of this modification, the tyrosine residue is highly evolutionarily conserved. A schematic outlining this model is presented in Supplementary Fig. 7.

Although Src alone is sufficient to phosphorylate ACF7 *in vitro*, presence of both FAK and Src is essential for optimal ACF7 phosphorylation in cells, reminiscent of the behaviour of other FAK/Src substrates involved in focal adhesion signalling^40^,^41^. In addition to forming a mutually activated kinase complex, FAK can also function as an adaptor molecule to allow physical association between Src and its substrates, such as endophilin and p130-Cas^40^,^41^. Association of ACF7 with microtubule plus tip complex has been well-characterized^7^, however, the potential interaction between ACF7 and focal adhesion molecules, such as FAK, is unclear. Thus, future investigation is required to further elucidate the molecular mechanism of how FAK and Src together mediate the phosphorylation of ACF7 during cell migration.

Tandem CH domains are present in many F-actin-interacting proteins and are well-known as binding motifs for F-actin^16^. Posttranslational modifications around the CH domains have been documented previously. The residue (tyrosine-12) in front of the first CH domain of α-actinin has been shown to be the target of FAK, and phosphorylation of this residue leads to diminished binding of α-actinin to F-actin^42^. In addition, it has been shown that different kinases can mediate phosphorylation of L-plastin at Serine 5 or 7 at the N terminus, and the modification can increase L-plastin association with F-actin^43^. However, all these modifications occur at residues outside the tandem CH domains, and it remains obscure how these modifications might affect the CH domains structurally.

Accumulating evidence suggests that the key ABSs of ABD are buried within tandem CH domains in the closed conformation. Thus, it has been speculated that certain conformational alteration is essential for the CH domains to expose the ABSs and associate with F-actin^16^,^26^,^30^, although different CH domain-bearing proteins may entail different mechanisms for their binding with F-actin^24^,^25^,^26^,^27^. Interestingly, the tyrosine 259 in ACF7 is conserved in several other CH domain-containing proteins (fimbrin and α-actinin). In this regard, it is also noteworthy that both fimbrin and α-actinin have been shown to localize at focal adhesions^44^,^45^. Thus, we posit that the FAK/Src-mediated phosphorylation could act as a general switch to regulate binding affinity of tandem CH domains, leading to recruitment of various F-actin-interacting proteins to cell-adhesion sites.

Tyrosine 259 localizes outside the three ABSs of ACF7’s CH domain, and our results suggest that phosphorylation of this residue can increase ACF7 association with F-actin by potentially relieving the intradomain interaction within ACF7’s ABD. This hypothesis is further supported by the mutagenesis results that mutation of two other isoleucine residues that contribute to the intradomain interaction can also increase the F-actin-binding affinity of ACF7 (Fig. 5d). However, based on our current results, we cannot completely rule out other potential mechanisms whereby phosphorylation of tyrosine 259 may contribute to F-actin binding of ACF7. For example, the modification may create an additional ABS for F-actin interaction in ACF7. Future study with solution structure and/or cryo-electron microscopy analysis will be essential to further define the molecular mechanism by which ACF7’s CH domains associate with F-actin, and how the interaction is regulated by phosphorylation of tyrosine 259.

In closing, our findings provide critical insights into the mechanics of cell-adhesion dynamics, and now pave the way for probing more deeply into the intricate signalling network orchestrating the crosstalk between F-actin and microtubule cytoskeletal networks at focal adhesions.

## Methods

### Reagents and plasmid DNA constructions

Rabbit serum against pan-ACF7 (dilution range 1:25–1:100) and phosphor-ACF7 Y259 (dilution range 1:50–1:200) was produced by Covance (Princeton, NJ) and Abmart (Shanghai, China), respectively. Guinea pig anti-K5 (dilution 1:200–1:500), rabbit anti-K10 (dilution 1:200–1:500) and Loricrin (dilution 1:200–1:500) antibodies were generous gifts from Dr Elaine Fuchs at the Rockefeller Univerisity. Rat monoclonal β4-integrin (CD104, BD 553,745, dilution 1:200–1:500) was obtained from BD Pharmingen (Franklin lakes, NJ). Human plasma fibronectin, ATP, HA-conjugated Agarose, mouse monoclonal Vinculin (Sigma V9131, dilution 1:200–1:500) and β-tubulin (Sigma T8328, dilution 1:200–1:500) Abs were obtained from Sigma (St Louis, MO). Texas Red-conjugated Phalloidin was obtained from Invitrogen (Carlsbad, CA). Mouse monoclonal Abs against Myc (Santa Cruz sc-40, dilution 1:500–1:2,000), and rabbit polyclonal Abs against HA (Santa Cruz sc-805, dilution 1:500–1:2,000), His tag (Santa Cruz sc-803, dilution 1:500–1:2,000), Src (Santa Cruz sc-19, dilution 1:500–1:1,000) and Ki67 (Santa Cruz sc-15402, dilution 1:500–1:1,000) and Src inhibitor pp2 were obtained from Santa Cruz Biotechnology (Santa Cruz, CA). FAK inhibitor PF-562271 was obtained from AdooQ Bioscience (Irvine, CA). Mouse monoclonal Antibody against phosphor-tyrosine was purchased from Millipore (Temecula, CA). Actin-binding protein spin-down kit was obtained from Cytoskeleton (Denver, CO). Other chemicals or reagents were obtained from Sigma, unless otherwise indicated.

ACF7 N-terminal (residues 1–400), CH domain (residues 1–268), plakin domain (residues 268–400) and their mutants were cloned into mammalian expression vectors pKH3S and pHANS (with N-terminal HA or Myc tag) and prokaryotic expression vector pETDuet1. Tyrosin259 (Y259) mutations to Phenylalanine (F) and Aspartic acid (D) in *ACF7-NT and ACF7 full length* were created by over-lapping PCRs with primers: 5′-CGCCTAGGTTTCCTGTTCTTTGGGCC-3′, 5′-CCGCTCGAGTTATTTGTTTGCAATCTGTAGCAG-3′, 5′-TGTGTCTTCGATTTTTGATGCCTTCCCTA-3′, 5′-TAGGGAAGGCATCAAAAATCGAAGACACA-3′, 5′-TGTGTCTTCGATTGATGATGCCTTCCCTA-3′, 5′-TAGGGAAGGCATCATCAATCGAAGACACA-3′. To create *ACF7*-YFSD mutant, the same mutagenesis PCR was carried out in *ACF7*-SD mutant background^14^. Full-length ACF7 and its mutant were cloned into PB vector p748-IRES-puro for construction of stable cell lines. Plasmid expressing transposase was kindly provided by Dr Jerrold Turner at the University of Chicago.

### Expression and purification of ACF7-NT

The ACF7-NT containing a 6 × His tag at the N terminus was overproduced in *Escherichia coli* BL21(DE3) cells. Briefly, cells were grown in 12 l of LB medium containing 100 μg ml^−1^ ampicillin at 30 °C. The culture was induced with 0.4 mM isopropyl-β,D-thiogalactopyranoside at an OD_600_ (optical density at 600 nm) value of ∼0.5. Cells were harvested within 5 h of induction. The collected bacteria were resuspended in ice-cold buffer containing 20 mM Na–HEPES (pH 7.5) and 100 mM NaCl. The cells were then lysed in a French pressure cell. Cell debris was removed by centrifugation for 1 h at 4 °C and 20,000*g*. ACF7-NT then purified with Ni^2+^-affinity and superdex-200 gel filtration (AKTA, GE healthcare Life Sciences, USA) columns to >95% purity and its identity was confirmed by N-terminal sequencing. ACF7-NT mutant (Y259D) was isolated with the same procedure.

### Purification of ACF7 and *in vitro* kinase assay

Full-length ACF7 was isolated by affinity chromatography from 293F clone that stably expresses HA-tagged *ACF7*^3^. For *in vitro* kinase assays, purified ACF7 or ACF7 mutants were incubated with recombinant kinases for 10 min at 37 °C in a total volume of 30 μl of protein kinase assay buffer (100 μM ATP, 10 mM HEPES at pH 7.4, 3 mM MgCl_2_, 3 mM MnCl_2_, 1 mM dithiothreitol, 10 mM β-mercaptoethanol)±1 μCi of [γ-^32^P] ATP (6,000 Ci mmol^−1^) (ref. 46).

### Microtubule-binding assay

Microtubule binding were examined using microtubule-binding protein spin-down assay kit from Cytoskeleton, according to manufacturer’s instructions. ACF7 truncation mutants were isolated from *E. coli* or expressed in HEK293T cells (Fig. 4c,d). For the latter, cell lysates were pre-cleaned by ultracentrifugation before the pull-down analysis.

### Crystallization and structural determination of ACF7-NT

Crystals of ACF7-NT were obtained using hanging-drop vapour diffusion at 4 °C. For ACF7-NT, a 1 μl 20 mg ml^−1^ ACF7-NT in a buffer containing 25 mM Hepes pH 7.5, 150 mM NaCl, 2 mM CaCl_2_ was mixed with 1 μl of reservoir solution containing 0.1 M MES pH 6.5, 0.2 M (NH_4_)_2_SO_4_ and 1 M (NH_4_)_2_HPO_4_. Cryoprotection was achieved by raising the glycerol concentration stepwise to 30% with a 5% increment in each step. The diffraction data sets of ACF7-NT were collected at 100 K at Shanghai Synchrotron Radiation Facility. Diffraction data were processed with DENZO and scaled with SCALEPACK. The ACF7-NT structure was determined by molecular replacement with Phaser in CCP4i (ref. 47) using known structures (PDB code=3f7p and 2odv, respectively) as the search model. After tracing the initial model manually in the program Coot^48^, the model was then refined against the native data at 2.6-Å resolution using CNS^49^ and PHENIX^50^. The structure data have been deposited to Protein Data Bank (PDB) with accession code 4Z6G.

### SAXS

SAXS data for ACF7-NT and ACF7-NT (Y259D) in PBS were collected at room temperature at 12ID-B, APS, ANL using 1 mg ml^−1^ protein and an incident X-ray wavelength of 0.886 Å. The data was reduced and analysed using ATSAS^51^. PRIMUS was used to determine the *R*_g_ value in reciprocal space^52^. The SAXS data was deposited to SASBDB with accession code SASDBL3 for ACF7-NT and SASDBN3 for ACF7-NT Y259D.

### Histology and immunofluorescence

Skin or wound samples were embedded in OCT, frozen, sectioned and fixed in 4% formaldehyde. For paraffin sections, samples were incubated in 4% formaldehyde at 4 °C overnight, dehydrated with a series of increasing concentrations of ethanol and xylene, and then embedded in paraffin. Paraffin sections were rehydrated in decreasing concentrations of ethanol and subjected to antigen unmasking in 10 mM Citrate, pH 6.0. Sections were subjected to haematoxylin and eosin staining or immunofluorescence staining^53^. Antibodies were diluted according to manufacturer’s instruction, unless indicated.

### Cell culture and protein analysis

Primary mouse keratinocytes were isolated from the epidermis of newborn mice using trypsin, after prior separation of the epidermis from the dermis by an overnight dispase treatment. Keratinocytes were plated on mitomycin C–treated 3T3 fibroblast feeder cells until passage 3. Cells were cultured in E-media supplemented with 15% serum and a final concentration of 0.05 mM Ca^2+^. All experiments were performed using primary cells with <10 passages. FAK KO keratinocytes were kindly provided by Dr Markus Schober from New York University. HEK293 cells were cultured in DMEM medium supplemented with 10% serum. For protein analysis, cells were lysed in lysis buffer and subjected to immunoprecipitation with different antibodies. Whole-cell lysates and immunoprecipitates were examined by western blotting analysis with Odyssey infared imaging system or ECL reagents. Uncropped original scan of western results are shown in Supplementary Fig. 8.

### Cell-migration assays and time-lapse videomicroscopy

For scratch-wound healing assay, keratinocytes were plated on 35-mm tissue culture dish coated with fibronectin^46^. After cells reached confluency, wounds were created by manual scraping of the cell monolayer with a pipette tip. The dishes were then washed with PBS, replenished with media and photographed using a phase contrast microscope. Afterwards, dishes were placed in the tissue culture incubator, and the matched wound regions were photographed 12 and 24 h after wounding.

To trace the movement of individual keratinocytes, cells were plated on fibronectin-coated dishes and imaged with an Olympus vivaview microscope (× 20) for 2 h at 1 frame per min and manually tracked in Image J.

### Focal adhesion assembly and disassembly measurements

Keratinocytes were plated on fibronectin-coated dishes and transfected with plasmid encoding DsRed-Zyxin. Time series of images were acquired on a 3i Marianas Yokogawa-type spinning-disc confocal microscope equipped with a × 100 α-plane (1.45 oil) lens and an electron microscopy charge-coupled device camera. The rate constants for focal adhesion assembly and disassembly were obtained by calculating the slope of relative fluorescence intensity decreases of individual focal adhesion on a semilogarithmic scale against time^22^,^33^.

### Skin engraftment and skin-wound healing

Decelluralized dermis (1 × 1 cm) was prepared by EDTA treatment of newborn mouse skin^22^. About 1.5 × 10^6^ cultured keratinocytes were seeded onto the dermis in cell culture insert. After overnight attachment, the skin culture was exposed to air/liquid interphase. To transplant to nude animals, two 1 × 1-cm wounds were introduced to the back skin. After transplantation, the wound edge is sealed with surgical glue. The grafted animals were housed separately and the wound bandages were usually removed 1 week post surgery. At 2 weeks post surgery, full-thickness excisional wounds were made on skin grafts using a 6-mm skin puncher. Mice were housed separately, and no self-induced trauma was observed in control or cKO mice. Tissue was collected 2–6 days after wounding, and wound re-epithelialization was evaluated by histological analyses. Hyperproliferative epidermis (HE) was identified by haematoxylin and eosin staining, and the length of HE that extended into the wounds was measured and quantified.

For skin explant, the punched skin biopsy was cultured on matrigel-coated coverslip with E-medium added with 0.3 mM CaCl2. Pictures were taken at different time points as indicated. The animal studies were carried out in the ALAAC-accredited animal research facility at the University of Chicago. All the animal experiments were approved by the Institutional Animal Care and Use Committee of the University of Chicago.

### Intravital imaging of mice

Optical imaging was performed in the integrated small animal imaging research resource (iSAIRR) at the University of Chicago. Bioluminescence images were acquired on an IVIS Spectrum (Caliper Life Sciences, Alameda, CA) after animal was injected with luciferin (100 mg kg^−1^). Acquisition and image analysis were performed with Living Image 4.3.1 software.

Wound healing in grafted skin was imaged by multiphoton microscope in the light microscopy centre at the University of Chicago. Images were analysed with Image J and Ibidi chemotaxis & migration softwares. Movement speed and directions were quantified by tracking the movement of H2B–RFP-positive cell nuclear. Persistence of cell movement was determined by comparing the Euclidian distance (the length of the straight line connecting the start and end points) versus accumulated distance of cell migration.

### Statistical analysis

Statistical analysis was performed using Excel or OriginLab software. Box plots are used to describe the entire population without assumptions on the statistical distribution. A student *t*-test or analysis of variance was used to assess the statistical significance (*P* value) of differences between experimental conditions.

### Data availability

All relevant data are available from the authors. The SAXS data was deposited to SASBDB with accession code SASDBL3 for ACF7-NT and SASDBN3 for ACF7-NT Y259D. The structure data has been deposited to Protein Data Bank (PDB) with accession code 4Z6G.

## Additional information

**Accession codes:** The SAXS data was deposited to SASBDB with accession code SASDBL3 for ACF7-NT and SASDBN3 for ACF7-NT Y259D. The structure data has been deposited to Protein Data Bank (PDB) with accession code 4Z6G.

**How to cite this article:** Yue, J. *et al*. *In vivo* epidermal migration requires focal adhesion targeting of ACF7. *Nat. Commun.* 7:11692 doi: 10.1038/ncomms11692 (2016).

## Supplementary Material

Supplementary Information Supplementary Figures 1-8

Supplementary Movie 1 Intravital imaging of WT epidermal cell migration *in vivo* (one frame per minute, 30 minutes).

Supplementary Movie 2 Intravital imaging of *ACF7* KO epidermal cell migration *in vivo* (one frame per minute, 30 minutes).

Supplementary Movie 3 Rescue of cytoskeletal coordination by exogenous expression of *ACF7*. KO keratinocytes stably rescued with *ACF7* were transfected with plasmids encoding GFP-EB1 (green, label microtubule plus-ends), CFP-LifeAct (blue, label F-actin), and tagRFP-Zyxin (red, focal adhesion). Cells were then examined by multi-channel confocal spinning-disc videomicroscopy to record coordinated cytoskeletal dynamics. Images were taken at 1 frame every 1.5 seconds

Supplementary Movie 4 Rescue of cytoskeletal coordination by exogenous expression of* ACF7-Y259F* mutant. KO keratinocytes stably rescued with *ACF7-Y259F* mutant were transfected with plasmids encoding GFP-EB1 (green, label microtubule plus-ends), CFP-LifeAct (blue, label F-actin), and tagRFP-Zyxin (red, focal adhesion). Cells were then examined by multi-channel confocal spinning-disc videomicroscopy to record coordinated cytoskeletal dynamics. Images were taken at 1 frame every 1.5 seconds

Supplementary Movie 5 Migration of ACF7 KO cells rescued with WT *ACF7* *in vivo* (one frame per minute, 30 minutes).

Supplementary Movie 6 Migration of ACF7 KO cells rescued with Y259F *ACF7* *in vivo* (one frame per minute, 30 minutes).

## Figures and Tables

**Table 1:** Data collection and refinement statistics.

	ACF7-NT
*Data collection*	
Space group	P43212
Cell dimensions	
*a, b, c* (Å)	94.09, 94.09, 192.63
*α*, *β*, *γ* (°)	90.00, 90.00, 90.00
Resolution (Å)	50.00–2.60
*R*_merge_ (%)	5.7 (35.6)*
*I/σ*	19.4 (2.1)
Completeness (%)	97.9 (95.8)
Redundancy	4.6 (4.3)*
	
*Refinement*	
Resolution (Å)	24.18–2.65
No. of reflections	25,589
*R*_work_/*R*_free_	20.30/23.60
No. atoms	
Protein	2,757
Ligand/ion	10
Water	24
B-factors	
Protein	40
Ligand/ion	20
Water	20
R.m.s deviations	
Bond lengths (Å)	0.008
Bond angles (°)	1.081

^*^The high resolution shell is 2.6.

## References

[b1] Lauffenburger D. A. & Horwitz A. F. Cell migration: a physically integrated molecular process. Cell 84, 359–369 (1996). 8608589 10.1016/s0092-8674(00)81280-5

[b2] Rodriguez O. C. . Conserved microtubule-actin interactions in cell movement and morphogenesis. Nat. Cell Biol. 5, 599–609 (2003). 12833063 10.1038/ncb0703-599

[b3] Wu X., Kodama A. & Fuchs E. ACF7 regulates cytoskeletal-focal adhesion dynamics and migration and has ATPase activity. Cell 135, 137–148 (2008). 18854161 10.1016/j.cell.2008.07.045 PMC2703712

[b4] Yue J. . Microtubules regulate focal adhesion dynamics through MAP4K4. Dev. Cell 31, 572–585 (2014). 25490267 10.1016/j.devcel.2014.10.025 PMC4261153

[b5] Jefferson J. J., Leung C. L. & Liem R. K. Plakins: goliaths that link cell junctions and the cytoskeleton. Nat. Rev. Mol. Cell Biol. 5, 542–553 (2004). 15232572 10.1038/nrm1425

[b6] Roper K., Gregory S. L. & Brown N. H. The ‘spectraplakins’: cytoskeletal giants with characteristics of both spectrin and plakin families. J. Cell Sci. 115, 4215–4225 (2002). 12376554 10.1242/jcs.00157

[b7] Suozzi K. C., Wu X. & Fuchs E. Spectraplakins: Master orchestrators of cytoskeletal dynamics. J. Cell Biol. 197, 465–475 (2012). 22584905 10.1083/jcb.201112034 PMC3352950

[b8] Chen H. J. . The role of microtubule actin cross-linking factor 1 (MACF1) in the Wnt signaling pathway. Genes Dev. 20, 1933–1945 (2006). 16815997 10.1101/gad.1411206 PMC1522081

[b9] Kodama A., Karakesisoglou I., Wong E., Vaezi A. & Fuchs E. ACF7: an essential integrator of microtubule dynamics. Cell 115, 343–354 (2003). 14636561 10.1016/s0092-8674(03)00813-4

[b10] Antonellis P. J. . ACF7 is a hair-bundle antecedent, positioned to integrate cuticular plate actin and somatic tubulin. J. Neurosci. 34, 305–312 (2014). 24381291 10.1523/JNEUROSCI.1880-13.2014 PMC3866489

[b11] Fassett J. T. . Microtubule Actin Cross-linking Factor 1 regulates cardiomyocyte microtubule distribution and adaptation to hemodynamic overload. PLoS ONE 8, e73887 (2013). 24086300 10.1371/journal.pone.0073887 PMC3784444

[b12] Goryunov D., He C. Z., Lin C. S., Leung C. L. & Liem R. K. Nervous-tissue-specific elimination of microtubule-actin crosslinking factor 1a results in multiple developmental defects in the mouse brain. Mol. Cell. Neurosci. 44, 1–14 (2010). 20170731 10.1016/j.mcn.2010.01.010 PMC2847646

[b13] Ka M., Jung E. M., Mueller U. & Kim W. Y. MACF1 regulates the migration of pyramidal neurons via microtubule dynamics and GSK-3 signaling. Dev. Biol. 395, 4–18 (2014). 25224226 10.1016/j.ydbio.2014.09.009 PMC4190130

[b14] Wu X. . Skin stem cells orchestrate directional migration by regulating microtubule-ACF7 connections through GSK3beta. Cell 144, 341–352 (2011). 21295697 10.1016/j.cell.2010.12.033 PMC3050560

[b15] Karakesisoglou I., Yang Y. & Fuchs E. An epidermal plakin that integrates actin and microtubule networks at cellular junctions. J. Cell Biol. 149, 195–208 (2000). 10747097 10.1083/jcb.149.1.195 PMC2175090

[b16] Sjoblom B., Ylanne J. & Djinovic-Carugo K. Novel structural insights into F-actin-binding and novel functions of calponin homology domains. Curr. Opin. Struct. Biol. 18, 702–708 (2008). 18952167 10.1016/j.sbi.2008.10.003

[b17] Hynes R. O. Integrins: bidirectional, allosteric signaling machines. Cell 110, 673–687 (2002). 12297042 10.1016/s0092-8674(02)00971-6

[b18] Brunton V. G. & Frame M. C. Src and focal adhesion kinase as therapeutic targets in cancer. Curr. Opin. Pharmacol. 8, 427–432 (2008). 18625340 10.1016/j.coph.2008.06.012

[b19] Huveneers S. & Danen E. H. Adhesion signaling—crosstalk between integrins, Src and Rho. J. Cell Sci. 122, 1059–1069 (2009). 19339545 10.1242/jcs.039446

[b20] Peng X. & Guan J. L. Focal adhesion kinase: from *in vitro* studies to functional analyses *in vivo*. Curr. Protein Pept. Sci. 12, 52–67 (2011). 21190526 10.2174/138920311795659452

[b21] Webb D. J. . FAK-Src signalling through paxillin, ERK and MLCK regulates adhesion disassembly. Nat. Cell Biol. 6, 154–161 (2004). 14743221 10.1038/ncb1094

[b22] Prunieras M., Regnier M. & Woodley D. Methods for cultivation of keratinocytes with an air-liquid interface. J. Invest. Dermatol. 81, 28s–33s (1983). 6190962 10.1111/1523-1747.ep12540324

[b23] Friedl P. & Gilmour D. Collective cell migration in morphogenesis, regeneration and cancer. Nat. Rev. Mol. Cell Biol. 10, 445–457 (2009). 19546857 10.1038/nrm2720

[b24] Broderick M. J., Bobkov A. & Winder S. J. Utrophin ABD binds to F-actin in an open conformation. FEBS Open Biol. 2, 6–11 (2012). 10.1016/j.fob.2012.01.001 PMC3642092 23650574

[b25] Galkin V. E., Orlova A., Cherepanova O., Lebart M. C. & Egelman E. H. High-resolution cryo-EM structure of the F-actin-fimbrin/plastin ABD2 complex. Proc. Natl Acad. Sci. USA 105, 1494–1498 (2008). 18234857 10.1073/pnas.0708667105 PMC2234172

[b26] Lin A. Y., Prochniewicz E., James Z. M., Svensson B. & Thomas D. D. Large-scale opening of utrophin’s tandem calponin homology (CH) domains upon actin binding by an induced-fit mechanism. Proc. Natl Acad. Sci. USA 108, 12729–12733 (2011). 21768337 10.1073/pnas.1106453108 PMC3150896

[b27] Singh S. M. & Mallela K. M. The N-terminal actin-binding tandem calponin-homology (CH) domain of dystrophin is in a closed conformation in solution and when bound to F-actin. Biophys. J. 103, 1970–1978 (2012). 23199925 10.1016/j.bpj.2012.08.066 PMC3491715

[b28] Jefferson J. J., Ciatto C., Shapiro L. & Liem R. K. Structural analysis of the plakin domain of bullous pemphigoid antigen1 (BPAG1) suggests that plakins are members of the spectrin superfamily. J. Mol. Biol. 366, 244–257 (2007). 17161423 10.1016/j.jmb.2006.11.036 PMC1850962

[b29] Ortega E., Buey R. M., Sonnenberg A. & de Pereda J. M. The structure of the plakin domain of plectin reveals a non-canonical SH3 domain interacting with its fourth spectrin repeat. J. Biol. Chem. 286, 12429–12438 (2011). 21288893 10.1074/jbc.M110.197467 PMC3069446

[b30] Moores C. A., Keep N. H. & Kendrick-Jones J. Structure of the utrophin actin-binding domain bound to F-actin reveals binding by an induced fit mechanism. J. Mol. Biol. 297, 465–480 (2000). 10715214 10.1006/jmbi.2000.3583

[b31] Di Matteo M. . PiggyBac toolbox. Methods Mol. Biol. 859, 241–254 (2012). 22367876 10.1007/978-1-61779-603-6_14

[b32] Schober M. . Focal adhesion kinase modulates tension signaling to control actin and focal adhesion dynamics. J. Cell Biol. 176, 667–680 (2007). 17325207 10.1083/jcb.200608010 PMC2064024

[b33] Riedl J. . Lifeact: a versatile marker to visualize F-actin. Nat. Methods 5, 605–607 (2008). 18536722 10.1038/nmeth.1220 PMC2814344

[b34] Mazzalupo S., Wawersik M. J. & Coulombe P. A. An *ex vivo* assay to assess the potential of skin keratinocytes for wound epithelialization. J. Invest. Dermatol. 118, 866–870 (2002). 11982766 10.1046/j.1523-1747.2002.01736.x

[b35] Kaverina I., Krylyshkina O. & Small J. V. Microtubule targeting of substrate contacts promotes their relaxation and dissociation. J. Cell Biol. 146, 1033–1044 (1999). 10477757 10.1083/jcb.146.5.1033 PMC2169483

[b36] Krylyshkina O. . Modulation of substrate adhesion dynamics via microtubule targeting requires kinesin-1. J. Cell Biol. 156, 349–359 (2002). 11807097 10.1083/jcb.200105051 PMC2199234

[b37] Bershadsky A., Chausovsky A., Becker E., Lyubimova A. & Geiger B. Involvement of microtubules in the control of adhesion-dependent signal transduction. Curr. Biol. 6, 1279–1289 (1996). 8939572 10.1016/s0960-9822(02)70714-8

[b38] Ezratty E. J., Partridge M. A. & Gundersen G. G. Microtubule-induced focal adhesion disassembly is mediated by dynamin and focal adhesion kinase. Nat. Cell Biol. 7, 581–590 (2005). 15895076 10.1038/ncb1262

[b39] Kapur M. . Calcium tips the balance: a microtubule plus end to lattice binding switch operates in the carboxyl terminus of BPAG1n4. EMBO Rep. 13, 1021–1029 (2012). 22995871 10.1038/embor.2012.140 PMC3492708

[b40] Ruest P. J., Shin N. Y., Polte T. R., Zhang X. & Hanks S. K. Mechanisms of CAS substrate domain tyrosine phosphorylation by FAK and Src. Mol. Cell. Biol. 21, 7641–7652 (2001). 11604500 10.1128/MCB.21.22.7641-7652.2001 PMC99935

[b41] Wu X., Gan B., Yoo Y. & Guan J. L. FAK-mediated src phosphorylation of endophilin A2 inhibits endocytosis of MT1-MMP and promotes ECM degradation. Dev. Cell 9, 185–196 (2005). 16054026 10.1016/j.devcel.2005.06.006

[b42] Izaguirre G. . The cytoskeletal/non-muscle isoform of alpha-actinin is phosphorylated on its actin-binding domain by the focal adhesion kinase. J. Biol. Chem. 276, 28676–28685 (2001). 11369769 10.1074/jbc.M101678200

[b43] Janji B. . Phosphorylation on Ser5 increases the F-actin-binding activity of L-plastin and promotes its targeting to sites of actin assembly in cells. J. Cell Sci. 119, 1947–1960 (2006). 16636079 10.1242/jcs.02874

[b44] Matsudaira P. The fimbrin and alpha-actinin footprint on actin. J. Cell Biol. 126, 285–287 (1994). 8034735 10.1083/jcb.126.2.285 PMC2200041

[b45] Sjoblom B., Salmazo A. & Djinovic-Carugo K. Alpha-actinin structure and regulation. Cell. Mol. Life Sci. 65, 2688–2701 (2008). 18488141 10.1007/s00018-008-8080-8 PMC11131806

[b46] Wu X., Suetsugu S., Cooper L. A., Takenawa T. & Guan J. L. Focal adhesion kinase regulation of N-WASP subcellular localization and function. J. Biol. Chem. 279, 9565–9576 (2004). 14676198 10.1074/jbc.M310739200

[b47] McCoy A. J. . Phaser crystallographic software. J. Appl. Crystallogr. 40, 658–674 (2007). 19461840 10.1107/S0021889807021206 PMC2483472

[b48] Emsley P. & Cowtan K. Coot: model-building tools for molecular graphics. Acta Crystallogr. D Biol. Crystallogr. 60, 2126–2132 (2004). 15572765 10.1107/S0907444904019158

[b49] Brunger A. T. . Crystallography & NMR system: A new software suite for macromolecular structure determination. Acta Crystallogr. D Biol. Crystallogr. 54, 905–921 (1998). 9757107 10.1107/s0907444998003254

[b50] Adams P. D. . PHENIX: building new software for automated crystallographic structure determination. Acta Crystallogr. D Biol. Crystallogr. 58, 1948–1954 (2002). 12393927 10.1107/s0907444902016657

[b51] Petoukhov M. V. . New developments in the ATSAS program package for small-angle scattering data analysis. J. Appl. Crystallogr. 45, 342–350 (2012). 25484842 10.1107/S0021889812007662 PMC4233345

[b52] Konarev P. V., Volkov V. V., Sokolova A. V., Koch M. H. J. & Svergun D. I. PRIMUS: a Windows PC-based system for small-angle scattering data analysis. J. Appl. Crystallogr. 36, 1277–1282 (2003).

[b53] Guasch G. . Loss of TGFbeta signaling destabilizes homeostasis and promotes squamous cell carcinomas in stratified epithelia. Cancer Cell 12, 313–327 (2007). 17936557 10.1016/j.ccr.2007.08.020 PMC2424201

