# Supplementary material for: In vivo epidermal migration requires focal adhesion targeting of ACF7
Source: Nat Commun. 2016 May 24;7:11692. doi: 10.1038/ncomms11692 (PMC5476826; doi:10.1038/ncomms11692)
Supplement: Supplementary Information — Supplementary Figures 1-8 [file ncomms11692-s1.pdf]

Supplementary Figure 1

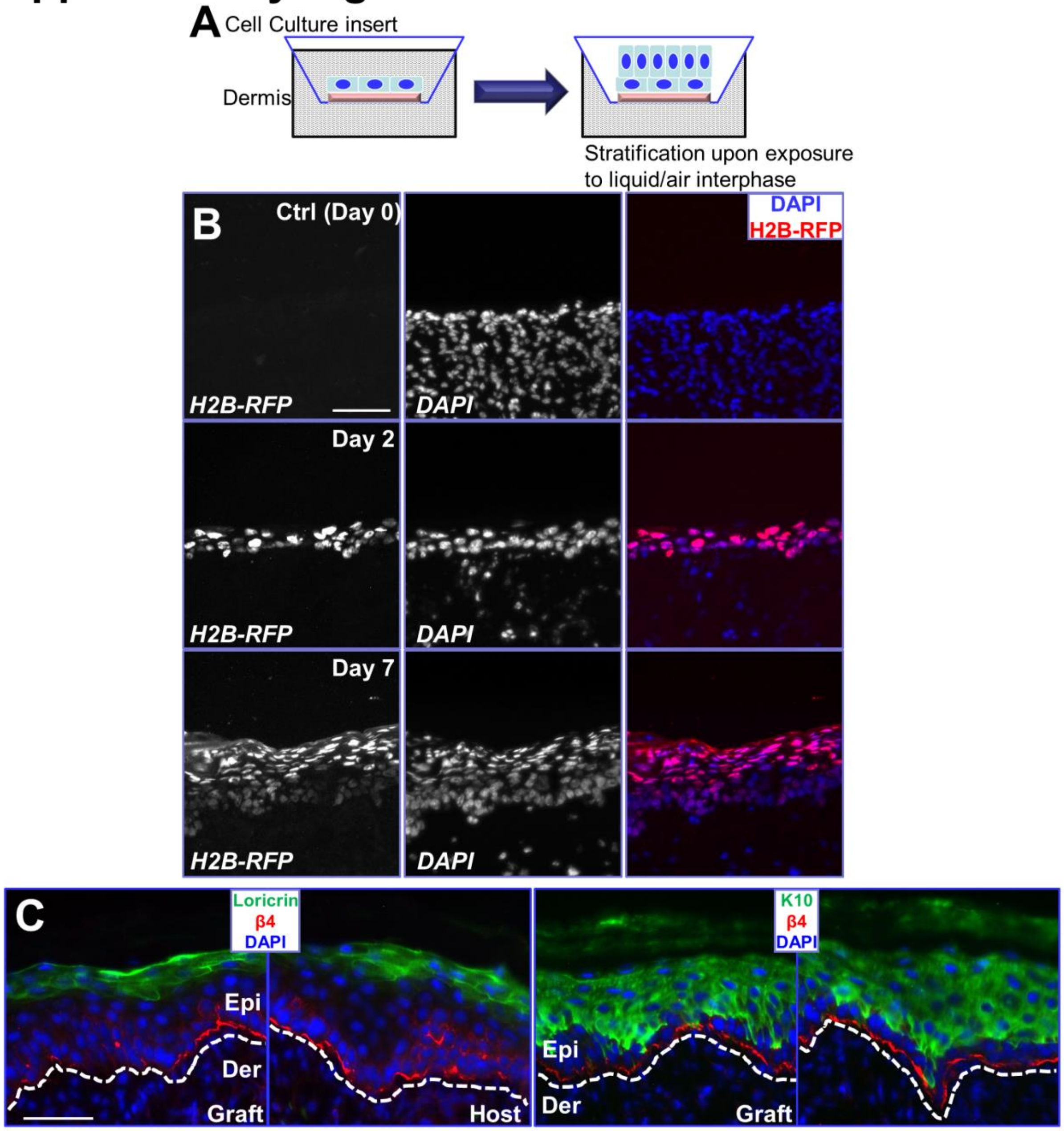

Supplementary Figure 1: Skin organotypic culture (related to Fig. 1)**. (A)** Diagram demonstrating the procedure for skin organotypic culture *in vitro*. Cultured skin keratinocytes were plated on top of acellularized dermis, and then exposed to air/liquid interphase to induce differentiation and stratification as skin epidermis *in vivo.* **(B)** Skin organotypic cultures were prepared with keratinocytes expressing *H2B-RFP* (red). Sections were prepared from skin culture at different time points as indicated, and stained with DAPI to show the cell nucleus (blue). Scale bar=50 μm. **(C)** Sections of

grafted skin and adjacent host skin were immunostained with different antibodies as indicated (K10: keratin 10, Lor: Loricrin, β4: β4-integrin, CD104). Dotted lines denote dermal–epidermal boundaries. Scale bar=50 μm.

Supplementary Figure 2

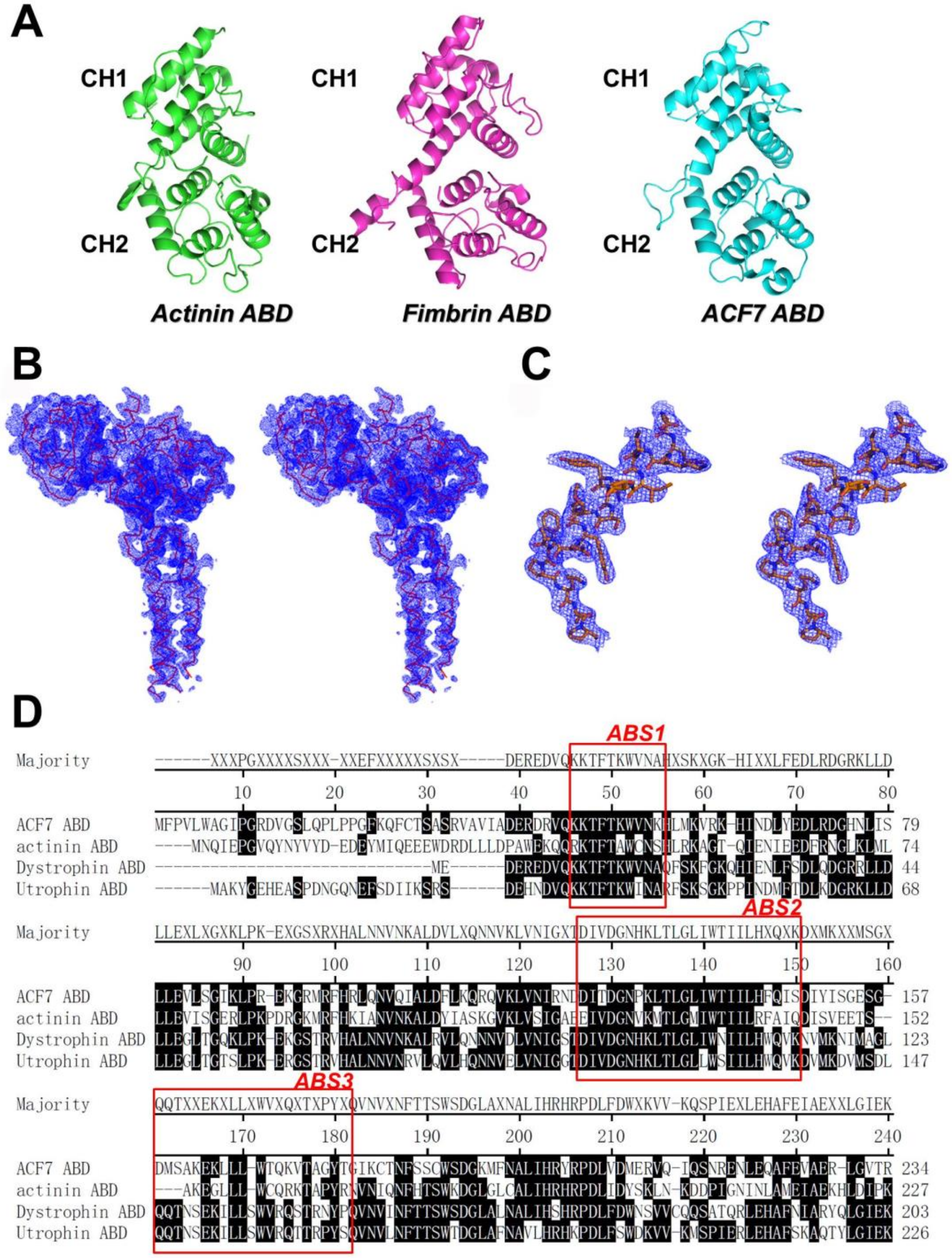

Supplementary Figure 2: Structural comparison of different tandem CH domains (related to Fig. 2)**. (A)** Structures of ABDs from Actinin, Fimbrin, and ACF7. Note closed conformation between CH1 and CH2. **(B-C)** Stereo view of the experimental electron density map at a resolution of 2.65 Å. (B) The

electron density map contoured at 1.2 σ is in blue. The C^{α} traces of molecule of ACF7-NT are in red. (C) Representative portion of the electron density in ACF7-NT. The electron density (colored blue) is contoured at the 1.2 σ level and superimposed with the final refined model (orange, carbon; red, oxygen; blue, nitrogen). **(D)** Sequence alignment of different ABDs from ACF7, actinin, dystrophin, and utrophin. Note the key actin binding sites (ABS1-3) are conserved among different proteins.

Supplementary Figure 3

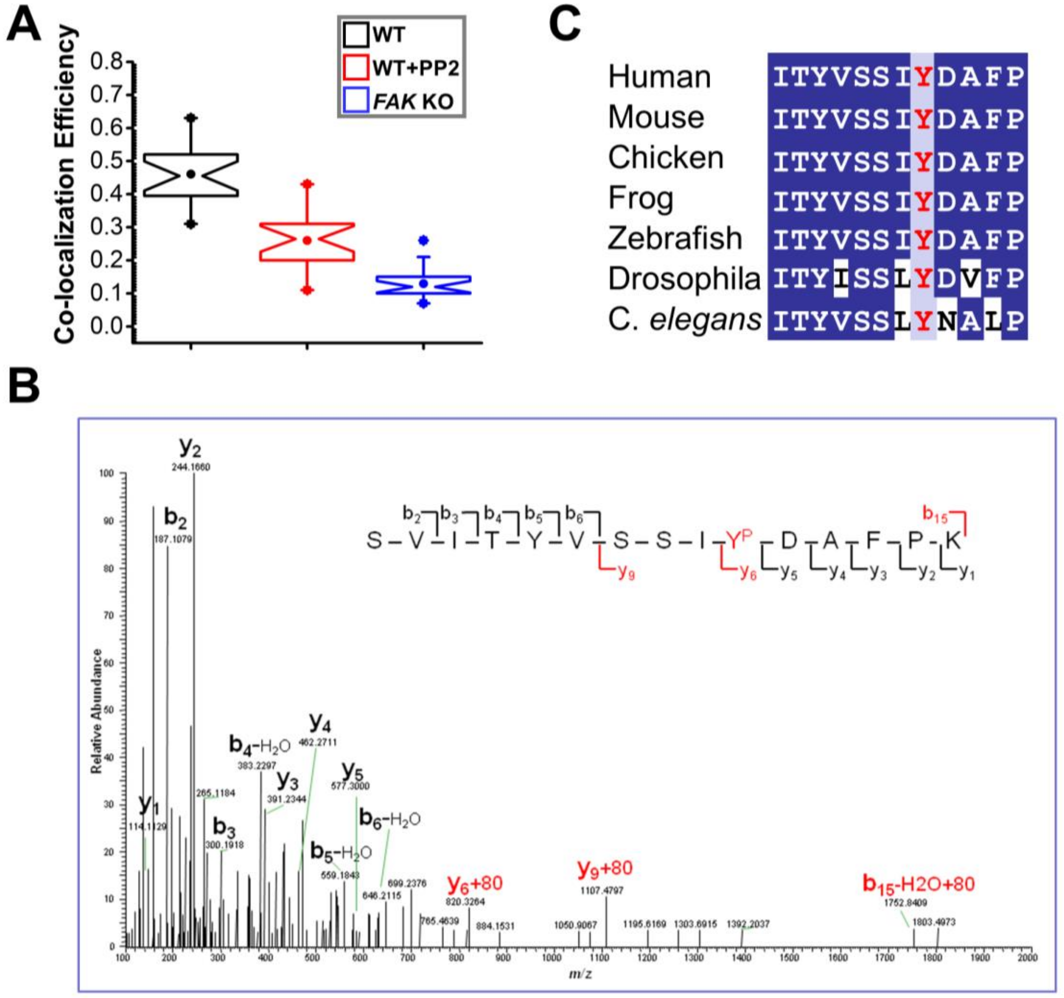

Supplementary Figure 3: FAK and Src phosphorylate ACF7's ABD (related to Fig. 3)**. (A)** Co-localization between ACF7 and focal adhesions (vinculin as marker) was determined by Pearson correlation coefficient. P<0.01 between WT and PP2 treated cells, or WT and *FAK* KO cells. **(B)** LC-MS/MS spectrum for identified peptide harboring phosphor-tyrosine 259. **(C)** Sequence alignment of different MACF1 proteins across species. Note the tyrosine residue (red) is evolutionarily conserved.

Supplementary Figure 4

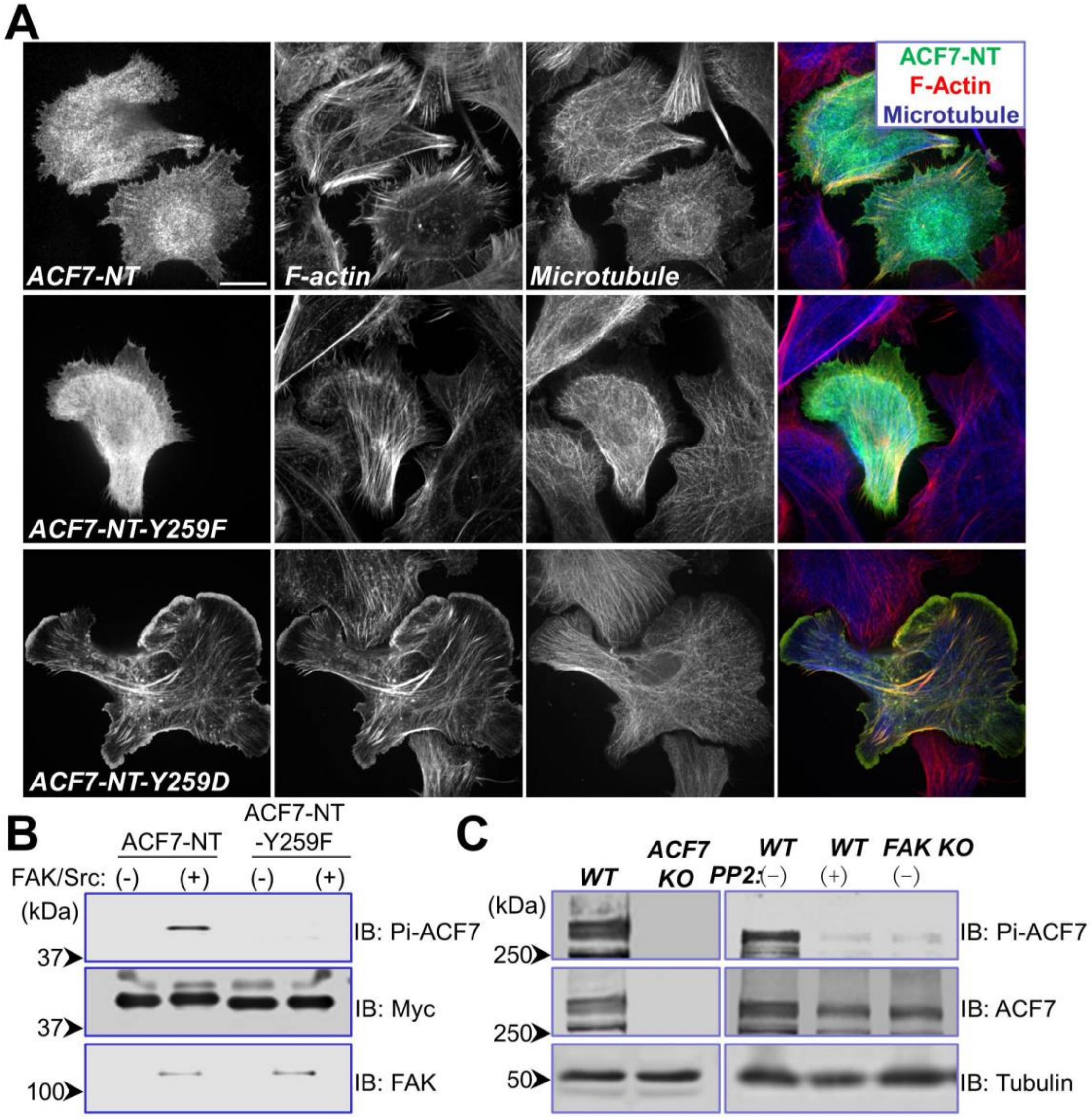

Supplementary Figure 4: Phosphorylation of ACF7 at Y259 regulates its F-actin binding affinity. (related to Fig. 4). **(A)** Keratinocytes were transfected with plasmids encoding *ACF7* NT or different NT mutants as indicated. Cells were subjected to immunofluorescence staining for ACF7 (green), F-actin (red), and microtubules (blue). Scale bar = 20 µm. **(B)** Lysates are collected from cultured cells expressing Myc-tagged ACF7-NT or ACF7-NT Y259F mutant together with or without FAK and Src. Lysates were analyzed by SDS-PAGE upon immunoprecipiation and immunoblotting with different antibodies as indicated. Pi-ACF7: ACF7 Y259 phospho-specific antibody. **(C)** Keratinocyte lysates

were prepared from WT keratinocytes, *ACF7* KO cell, FAK KO cells, or WT keratinocytes treated with Src inhibitor PP2. Lysates were subjected to immunoblot with ACF7 phospho-specific antibody and other antibodies as indicated.

Supplementary Figure 5

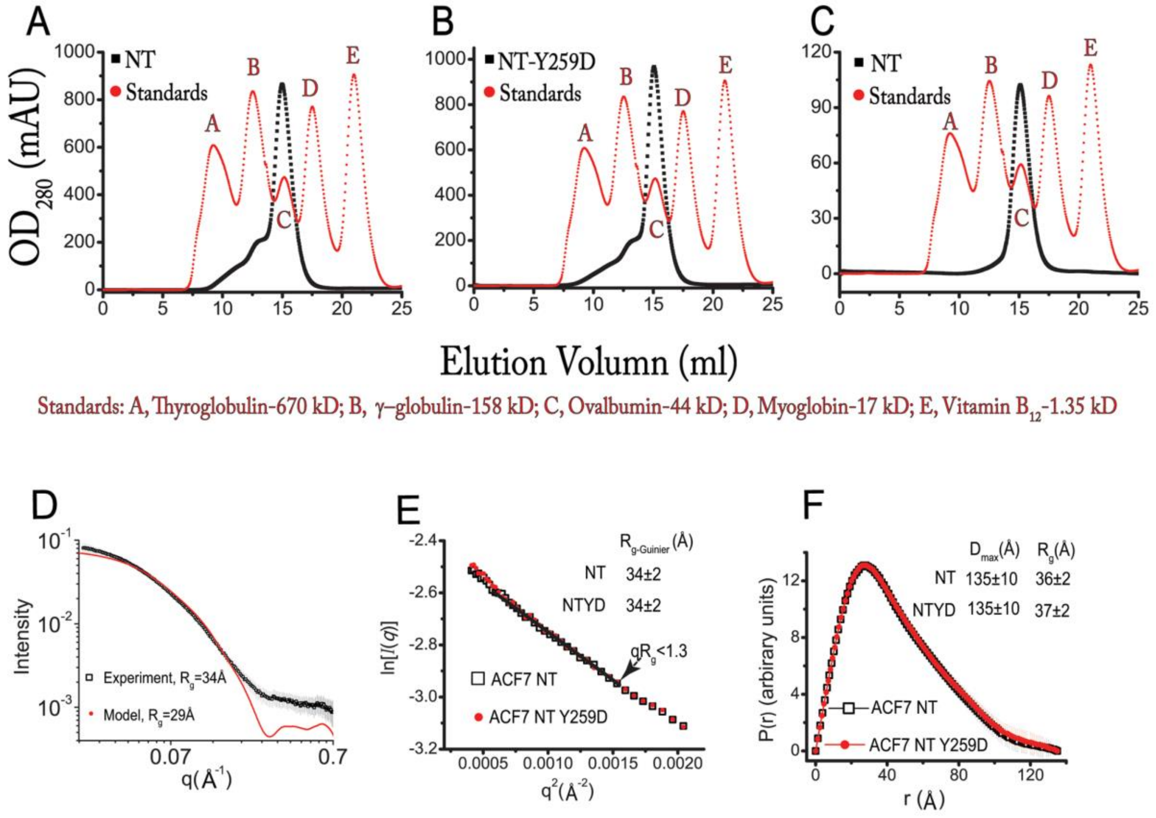

Supplementary Figure 5: SAXS profiles of ACF7 NT and NT Y259D in solution. (A,B) Profile of size exclusion column (Superdex 200 10/300 GL) of purified ACF7 NT and ACF7 NT Y259D shows that the proteins are monomeric and monodisperse. The profile of molecular weight standards (Bio-Rad) was colored in red. The peak of ACF7 NT (MW, 46.3 kD) overlaps with ovalbumin (44kD). This indicates that the main peaks of ACF7 NT and Y259D are monomer. We used such peaks of ACF7 NT and ACF7 NT Y259D for SAXS analysis, which is mono-disperse exemplified by the profile of ACF7 NT shown in **(C)**. **(D)** Comparison between experimental SAXS profile and the theoretical scattering curve of ACF7 NT**. (E)** The plots for obtaining R_g values of ACF7 NT and ACF7 NT Y259D by the Guinier approximation. **(F)** The pair distance distribution function of ACF7 NT and ACF7 NT Y259D. The calculation was done using PRIMUS an d GNOM in ATSAS.

Supplementary Figure 6

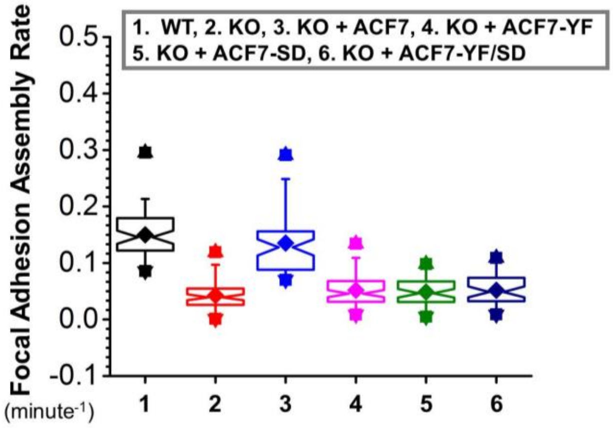

Supplementary Figure 6: Cytoskeletal crosslinking mediated by ACF7 promotes focal adhesion assembly. Box and whisker plots of focal adhesion assembly in different keratinocyte cell lines as indicated. One way ANOVA indicates that the difference between WT vs. KO, or KO vs. KO + ACF7, or KO + ACF7 vs. KO + ACF7-YF, or KO + ACF7 vs. KO + ACF7-SD, or KO + ACF7 vs. KO + ACF7-YFSD, is statistically significant ($P<0.05$).

Supplementary Figure 7

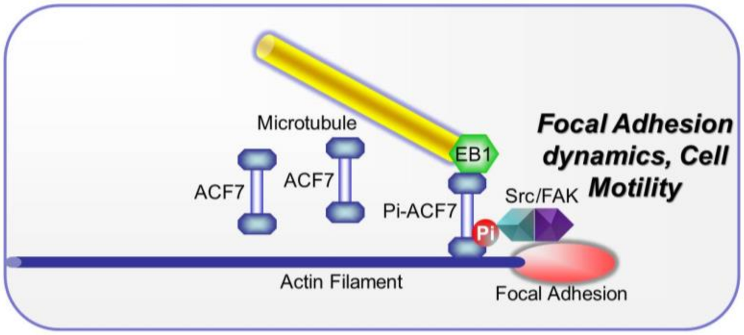

Supplementary Figure 7: A working model summarizing the role of FAK/Src mediated phosphorylation of ACF7 in focal adhesion turnover and cell migration. We posit that unmodified ACF7 cannot associate with F-actin. Activated FAK/Src tyrosine kinase complex at focal adhesions can phosphorylate ACF7 at Y259 in the ABD. The phosphorylation will relieve the intramolecular interactions within ACF7's tandem CH domains and enhance ACF7 association with F-actin. The specific association of ACF7 and F-actin at focal adhesions allow efficient targeting of microtubule plus ends toward focal adhesions via ACF7's microtubule and F-actin crosslinking activity, which promotes focal adhesion dynamics (assembly and disassembly) and cell motility. Pi-ACF7: ACF7 phosphorylated by FAK/Src.

Supplementary Figure 8

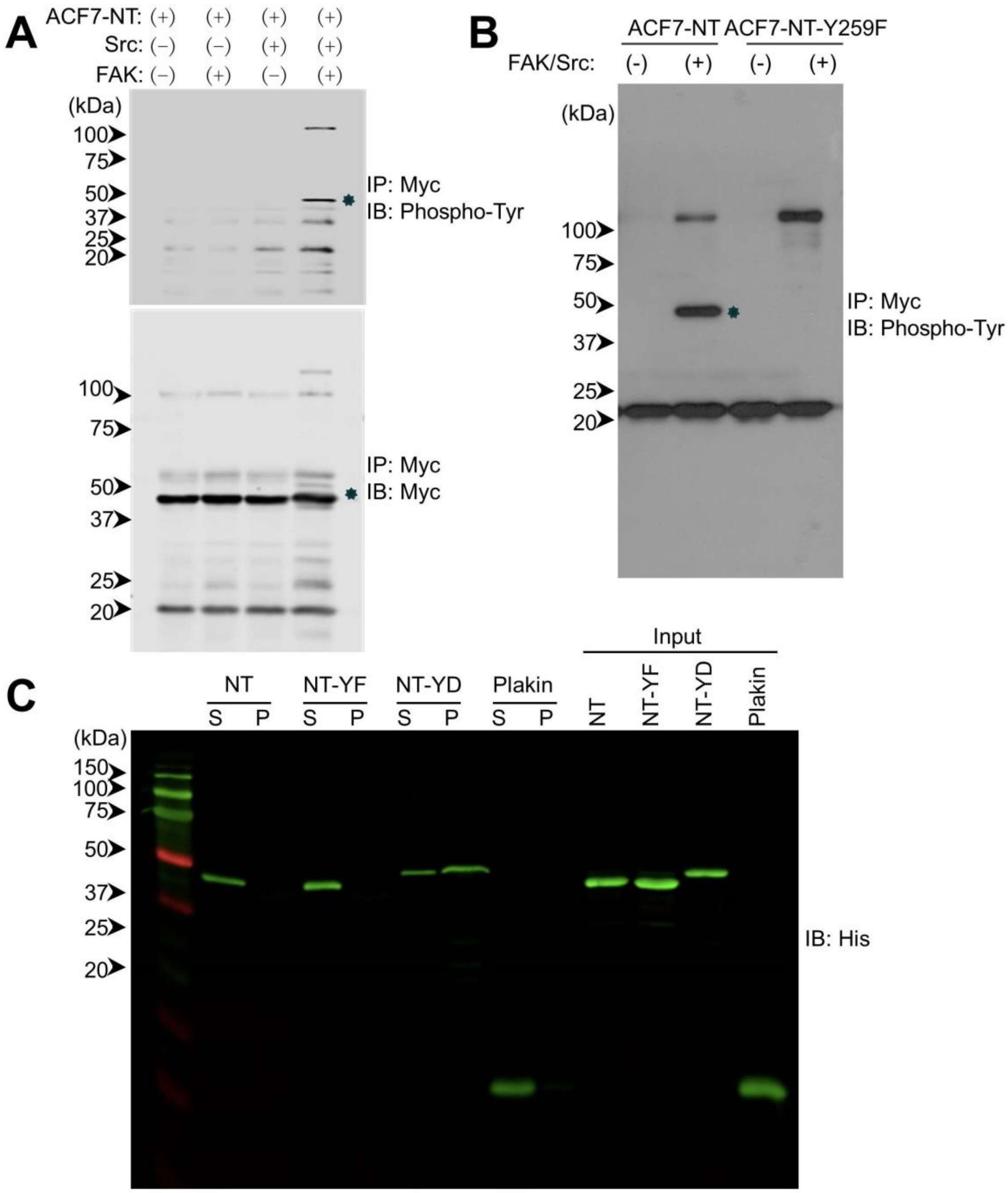

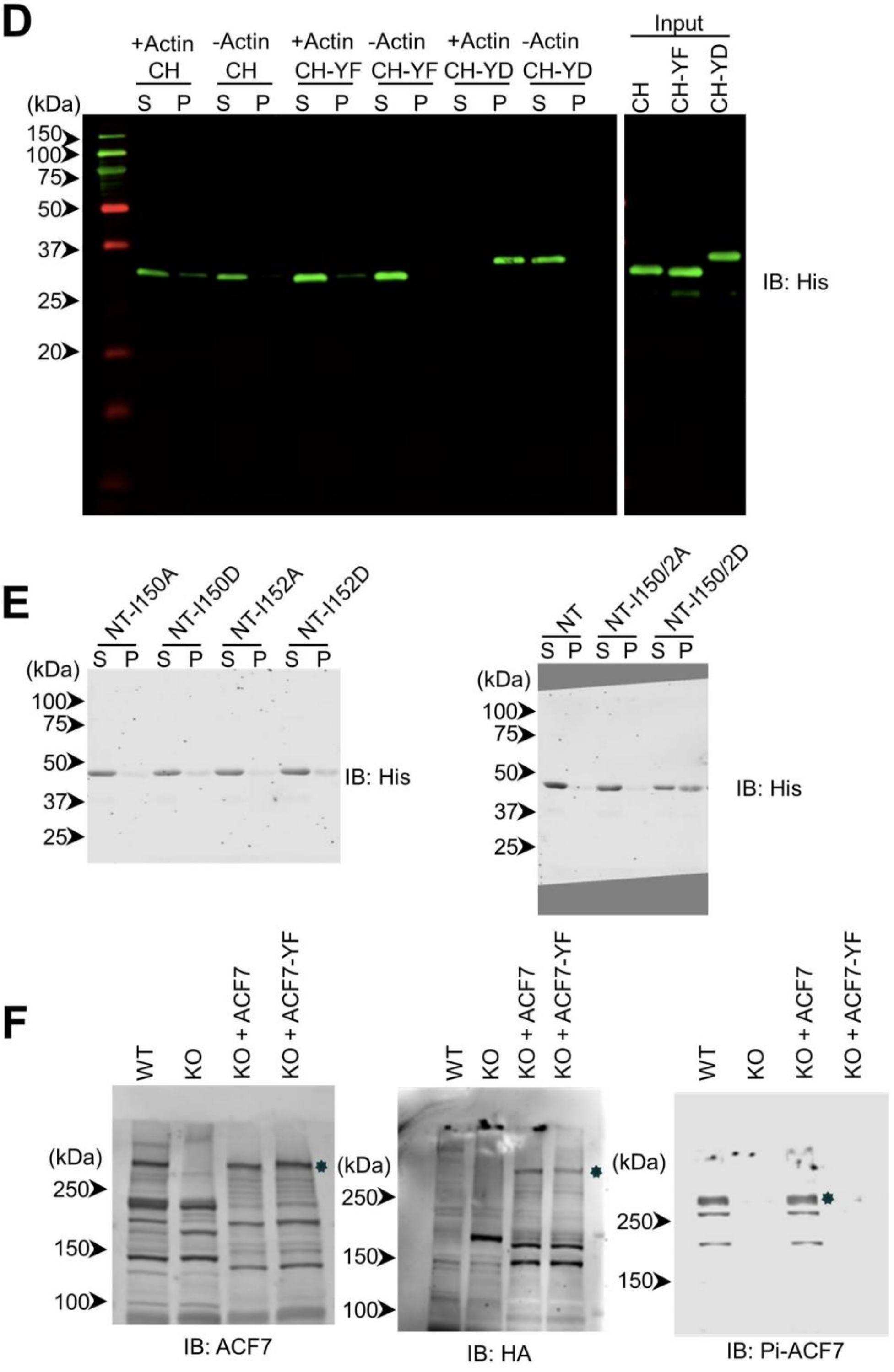

Supplementary Figure 8: Uncropped scans of western blots. **(A)** Uncropped scans for Figure 3C. The * denotes the bands for ACF7-NT. **(B)** Uncropped scans for Figure 3E. The * denotes the bands

for ACF7-NT. **(C)** Uncropped scans for Figure 4A. **(D)** Uncropped scans for Figure 4B. **(E)** Uncropped scans for Figure 5D. **(F)** Uncropped scans for Figure 6A. The * denotes the bands for full length ACF7.
